# Mobile App (WHEELS) to Promote a Healthy Lifestyle in Wheelchair Users With Spinal Cord Injury or Lower Limb Amputation: Usability and Feasibility Study

**DOI:** 10.2196/24909

**Published:** 2021-08-09

**Authors:** Dirk Hoevenaars, Jasmijn F M Holla, Leonie te Loo, Johan M Koedijker, Sarah Dankers, Han Houdijk, Bart Visser, Thomas W J Janssen, Sonja de Groot, Marije Deutekom, WHEELS Study Group

**Affiliations:** 1 Faculty of Behavioural and Movement Sciences Vrije Universiteit Amsterdam Netherlands; 2 Amsterdam Rehabilitation Research Center Reade Amsterdam Netherlands; 3 Faculty of Health, Sports and Social Work Inholland University of Applied Sciences Haarlem Netherlands; 4 Center for Adapted Sports Amsterdam Amsterdam Institute of Sport Science Amsterdam Netherlands; 5 Faculty of Health Amsterdam University of Applied Sciences Amsterdam Netherlands; 6 Center for Human Movement Sciences University Medical Center Groningen University of Groningen Groningen Netherlands; 7 Department of Research and Development Heliomare Rehabilitation Center Wijk aan Zee Netherlands; 8 Faculty of Sports and Nutrition Amsterdam University of Applied Sciences Amsterdam Netherlands; 9 see Acknowledgments

**Keywords:** mHealth, mobile app, lifestyle, usability, feasibility, wheelchair users, spinal cord injury, lower limb amputation

## Abstract

**Background:**

Maintaining a healthy lifestyle is important for wheelchair users’ well-being, as it can have a major impact on their daily functioning. Mobile health (mHealth) apps can support a healthy lifestyle; however, these apps are not necessarily suitable for wheelchair users with spinal cord injury or lower limb amputation. Therefore, a new mHealth app (WHEELS) was developed to promote a healthy lifestyle for this population.

**Objective:**

The objectives of this study were to develop the WHEELS mHealth app, and explore its usability, feasibility, and effectiveness.

**Methods:**

The WHEELS app was developed using the intervention mapping framework. Intervention goals were determined based on a needs assessment, after which behavior change strategies were selected to achieve these goals. These were applied in an app that was pretested on ease of use and satisfaction, followed by minor adjustments. Subsequently, a 12-week pre-post pilot study was performed to explore usability, feasibility, and effectiveness of the app. Participants received either a remote-guided or stand-alone intervention. Responses to semistructured interviews were analyzed using content analysis, and questionnaires (System Usability Score [SUS], and Usefulness, Satisfaction, and Ease) were administered to investigate usability and feasibility. Effectiveness was determined by measuring outcomes on physical activity, nutrition, sleep quality (Pittsburgh Sleep Quality Index), body composition, and other secondary outcomes pre and post intervention, and by calculating effect sizes (Hedges *g*).

**Results:**

Sixteen behavior change strategies were built into an app to change the physical activity, dietary, sleep, and relaxation behaviors of wheelchair users. Of the 21 participants included in the pilot study, 14 participants completed the study. The interviews and questionnaires showed a varied user experience. Participants scored a mean of 58.6 (SD 25.2) on the SUS questionnaire, 5.4 (SD 3.1) on ease of use, 5.2 (SD 3.1) on satisfaction, and 5.9 (3.7) on ease of learning. Positive developments in body composition were found on waist circumference (*P*=.02, *g*=0.76), fat mass percentage (*P*=.004, *g*=0.97), and fat-free mass percentage (*P*=.004, *g*=0.97). Positive trends were found in body mass (*P*=.09, *g*=0.49), BMI (*P=*.07, *g*=0.53), daily grams of fat consumed (*P*=.07, *g*=0.56), and sleep quality score (*P*=.06, *g*=0.57).

**Conclusions:**

The WHEELS mHealth app was successfully developed. The interview outcomes and usability scores are reasonable. Although there is room for improvement, the current app showed promising results and seems feasible to deploy on a larger scale.

## Introduction

A healthy lifestyle is known to be beneficial for a person’s well-being and happiness in many ways [1]. Healthy lifestyle behavior is especially important for wheelchair users owing to its major impact on their daily level of functioning [2]. Nevertheless, physical inactivity, obesity, and low vitality are common among wheelchair users with spinal cord injury (SCI) or lower limb amputation (LLA), which increase the risk of secondary health problems such as cardiovascular disease and can cause a reduced quality of life [3-9]. Therefore, it is important for wheelchair users to be encouraged to achieve and maintain a healthy lifestyle during and after inpatient rehabilitation [10-13].

Despite encouragement during inpatient rehabilitation, it appears to be difficult for wheelchair users with SCI or LLA to adopt or maintain the recommended physical activity levels and healthy diet after discharge [12,14-16]. Environmental factors such as fitness centers not having accessible toilets and personal factors such as lack of knowledge and motivation can play a role in this lack of physical activity. One of the barriers for maintaining a healthy lifestyle is the lack of professional guidance after discharge. Wheelchair users with SCI or LLA clearly express their need for such support [17,18], and guided interventions have shown positive effects for behavioral change and maintenance [19-21]. To save costs and time, mobile health (mHealth) tools could be used to support the professional in providing this additional guidance, which has been shown to be an effective method for changing behavior [22]. mHealth tools focus mainly on personal determinants of behavior. Although they cannot remove existing barriers in the physical or societal environment, they can increase the knowledge and skills to cope with these barriers.

mHealth provides the opportunity to stimulate, support, and monitor a healthy lifestyle at the individual and group levels [11]. Given that smartphones have become an integral part of our lives, mHealth seems to be a promising tool supporting healthy changes in physical activity, sedentary behavior, diet, relaxation, and sleep. A healthy lifestyle refers to the combination of healthy physical activity with appropriate dietary, relaxation, and sleep behaviors. Successful self-management apps have been developed for people with chronic conditions [23,24]. People tend to value mHealth apps that support goal-setting, and provide information and advice, feedback, self-monitoring tools, social support, and reinforcement [25]. The use of an appropriate combination of techniques to change lifestyle-related determinants mediates the potential effectiveness of an mHealth app [26,27]. However, determinants of physical activity, nutrition, and sleep can vary among populations and are different in individuals with a disability [28]. To date, there is no mHealth app designed specifically for wheelchair users with SCI or LLA to target all of these behaviors simultaneously. Therefore, in the Wheelchair ExercisE and Lifestyle Study (WHEELS) project, an existing lifestyle app for healthy able-bodied people was adapted for wheelchair users with SCI and LLA based on the intervention mapping protocol [29].

Targeting behavior change specifically for wheelchair users includes overcoming additional social barriers, which was taken into account during development of the WHEELS app [30]. An intervention targeting the combination of physical activity and dietary behavior seems to be superior to an intervention targeting physical activity or diet alone in weight management and improving health [31,32]. Because poor sleep quality and lack of sleep have a negative association with weight regulation, it seems to be of added value to also target resting and sleep behavior [33-35]. Given the positive relations between physical activity, diet, and sleep behavior, as well as combining multiple healthy lifestyle features, a lifestyle app was designed in which physical activity, dietary, sleep, and resting behaviors were targeted simultaneously [35-37].

To evaluate this combined lifestyle app, a usability and effectiveness study was performed in wheelchair users with SCI or LLA. A multicomponent intervention, which is a combination of different intervention components such as an app combined with counseling, seems to be more effective than a stand-alone app intervention [25]. This raised the question as to whether this would also be the case among wheelchair users. Therefore, both a stand-alone and a remote-guided version of the mHealth intervention were applied during the intervention period. In the remote-guided version, personal guidance from a lifestyle coach was offered throughout the intervention period. The aims of this study were to: (1) describe the development of the WHEELS mHealth app; and (2) explore its usability, feasibility, and effectiveness.

## Methods

### Development of the mHealth Intervention Using Intervention Mapping

The WHEELS lifestyle app was developed using the intervention mapping framework for planning theory- and evidence-based health promotion programs [38]. This framework consists of six steps: (1) perform a needs assessment and state intervention goals; (2) construct matrices of change objectives; (3) choose theory- and evidence-based behavior change methods and practical applications to deliver them; (4) pretest, refine, and produce the program; (5) develop an implementation plan; and (6) create an evaluation plan. These six intervention mapping steps to plan a mobile lifestyle intervention for wheelchair users with SCI or LLA, focusing particularly on steps 1 to 4, are presented in detail in Multimedia Appendix 1.

### App Description and Content

**Figure 1:**
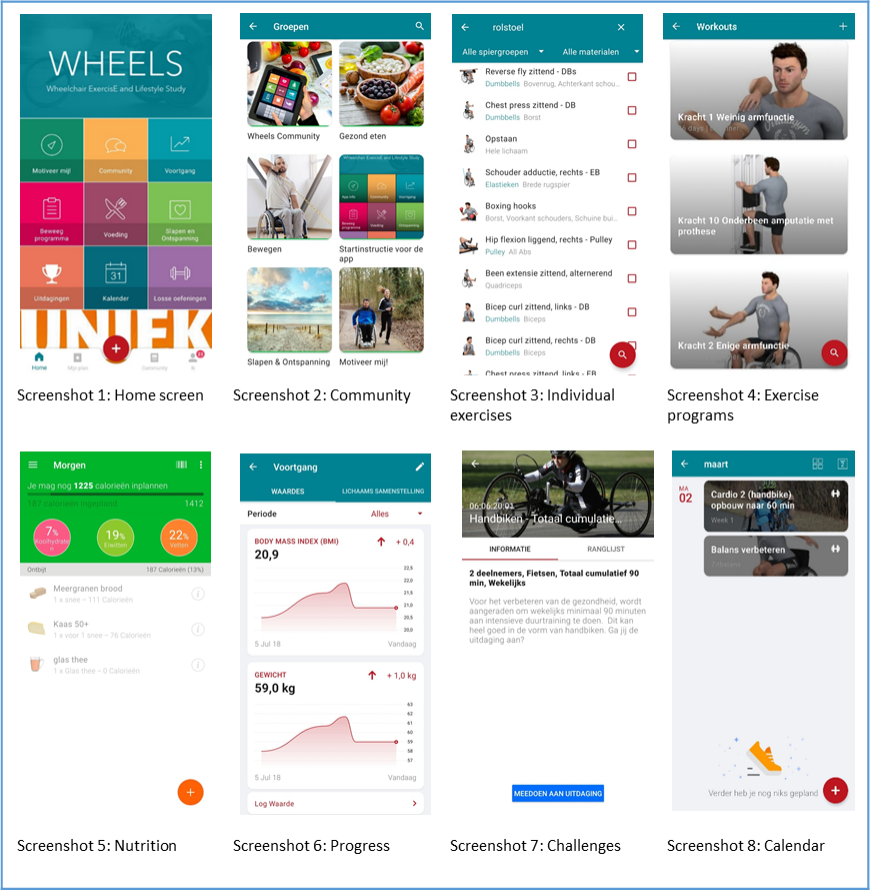
Structure overview of the WHEELS app.

The WHEELS app is targeted toward wheelchair users with SCI or LLA to help them comply with the scientific exercise guidelines for adults with SCI [7], achieve a healthy energy balance, and achieve a healthy balance between exercise and sleep/relaxation. In the app, wheelchair users are guided to these intervention goals by providing them knowledge and a format for setting personally meaningful subgoals and the functionalities described below. To work with the app, the user creates an account that they can personalize. Personal characteristics are used to provide feedback within the app (eg, height, body mass, and activity level are used to estimate resting energy expenditure). When logging in, users can navigate through the app from the home screen as shown in screenshot 1 in Figure 1. From the home screen, users can navigate to the “Individual exercises” and “Exercise program” tiles where exercises can be performed and scheduled in their personal agenda (Figure 1, screenshots 3 and 4). In the “Food” part, users receive an overview of their energy balance based on their nutrition intake and energy expenditure (Figure 1, screenshot 5). Nutrition plans and goals can be created (eg, losing weight) in which the app would guide the user through some steps toward a reasonable nutrition plan, resulting in a suggested daily energy intake. In the “Sleep & Relaxation” environment, exercises and knowledge are offered on balancing physical and mental load and relaxation. Behind the “Progress” tile, users are able to obtain insight and track changes in predefined health and fitness parameters such as weight and BMI. In addition, the “Community” section includes start instructions and four groups (exercise, nutrition, sleep and relaxation, lifestyle change tool) in which information and tips are posted over time (Figure 1, screenshot 2). A fifth group allows users to ask questions, share experiences and tips, and interact with other users. Finally, users are able join various challenges in which they can compare performances with each other (eg, a weekly 90-minute handcycle challenge) (Figure 1, screenshot 7).

### Usability and Feasibility Study

#### Participants

To evaluate the usability and feasibility of the app, potential end users were invited to participate in the study. Recruitment took place by advertisement at patient associations gatherings, on social media, and within the rehabilitation centers Reade (Amsterdam, the Netherlands) and Heliomare (Wijk aan Zee, the Netherlands). Potential participants were included when all of the following criteria were met: chronic SCI (including spina bifida) or LLA (>1 year), wheelchair-dependent for longer (>500 m) distances, 18 years or older, sufficient knowledge of the Dutch language, and access to a smartphone or tablet connected to the internet. Potential participants were excluded when one of the following criteria was met: insufficient understanding of technology to benefit from the app; limited functioning in the arm/hand to operate a smartphone or tablet; presence of progressive disorders that can influence the outcomes; presence of psychiatric disorders; and negative outcome to unsupervised exercise based on a medical screening, including a graded exercise test. The target was to include 15 individuals with SCI and 15 individuals with LLA resulting in a sufficient sample size with a possible 10% dropout rate, based on the literature [39]. All participants provided written informed consent and ethical approval was granted by the local Medical Ethical Committee of Slotervaart Ziekenhuis-Reade (METC nr. P1761).

#### Study Design and Protocol

**Figure 2:**
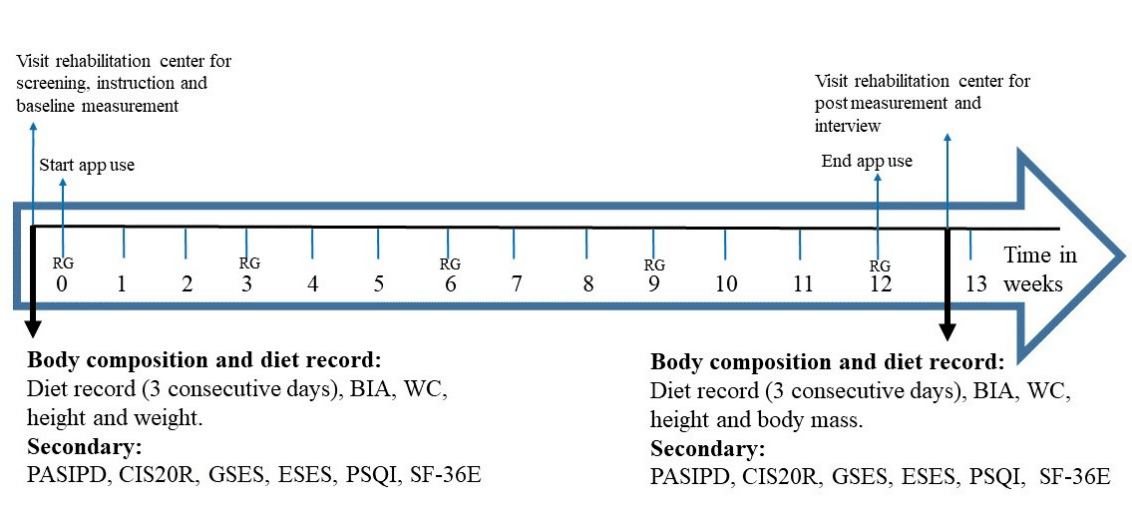
Schematic overview of measurements. BIA: bioimpedance analysis; CIS20R: Checklist Individual Strength; ESES: Exercise Self-Efficacy Scale for spinal cord injury; GSES: General Self-Efficacy Scale; PASIPD: Physical Activity Scale for Individuals with Physical Disabilities; PSQI: Pittsburgh Sleep Quality Index; RG: remote guidance; SF-36E: Short-Form 36 health survey; WC: waist circumference.

Participants were asked to participate in a 12-week pre-post pilot study focusing on the usability and feasibility of the app. Block randomization stratified by disorder with a block size of one was used to equally allocate the participants to a stand-alone or remote-guided intervention group. The remote-guided group received guidance from a lifestyle coach during the 12-week intervention (Figure 2). At the start of the study, the stand-alone group received an individual explanation and demonstration of the app from the researcher. During the study, this group was allowed to consult the researcher with any questions or difficulties regarding use of the app. The remote-guided group also received support at the start of the study, but additionally received remote guidance, consisting of an additional face-to-face consultation (30 minutes) at the start of the intervention and 10-15 minute contact moments after 3, 6, 9, and 12 weeks by phone, app, or email. The purpose of these contact moments was to discuss progress and to adjust the goals or the program if necessary. The two lifestyle coaches providing the supervision were 4th year students in Functional Exercise Therapy who were trained in motivational interviewing, assisted by an experienced rehabilitation professional from Reade or Heliomare who they could consult with any questions.

Owing to the design and nature of the study, it was not possible to blind participants or researchers. The participants kept a 3-day diet record during the same weeks as the scheduled pre and post measurements, which took place at a rehabilitation center. In addition, body composition was measured before and after the intervention, and participants were invited through email to complete an online questionnaire in Qualtrics to measure lifestyle and health-related quality of life. During the last visit, a semistructured interview was conducted and recorded.

#### Qualitative Evaluation: Usability and Feasibility

Topics evaluated to assess usability were: (1) usefulness, (2) ease of use, (3) satisfaction, (4) ease of learning, (5) motivation, (6) adherence, and (7) goals. In addition, semistructured interviews and questionnaires (System Usability Scale [SUS] [40], and Usefulness, Satisfaction, and Ease of Use [USE] [41]) were used to gain insight into usability and feasibility.

The interviews were conducted by the same two researchers and lecturers in Sports Studies (LtL and JK) who instructed the participants on how to use the app at the start of the study. The interviews took place following the postintervention measurements in the rehabilitation center in a private room, lasting on average 30 minutes and were audio-recorded with consent of the participants. An interview guide, developed by the research team and partially based on the SUS and USE questionnaires and broader literature on behavior change [41-43], was used to structure the interview. After starting with the question “How experienced are you with using smartphone apps?” the following topics were discussed: goals, adherence, motivation, ease of use, satisfaction, and usability. The complete interview guide is provided in Multimedia Appendix 2.

The SUS questionnaire is a 10-item Likert scale providing an overall subjective assessment on usability of a product. An 11-point Likert scale was used with a score ranging from 0 (“strongly disagree”) to 10 (“strongly agree”). A total score was calculated by rescaling the score to a total of 100, with a higher score indicating higher perceived usability [40]. A SUS score of 70 is considered to be average based on a wide range of interfaces [44].

Additionally, three out of four dimensions of the USE questionnaire were used to gain insight in the dimensions “ease of use,” “ease of learning,” and “satisfaction.” Each dimension is composed of 11, 4, and 7 items, respectively. The dimension “usefulness” was left out as it overlapped strongly with the SUS questionnaire. An 11-point Likert scale was used with a score ranging from 0 (“strongly disagree”) to 10 (“strongly agree”) and averaged for each dimension [41].

#### Quantitative Evaluation

##### Nutritional Habits

The diet record took place on 3 consecutive days with one weekend day, which is considered one of the most reliable methods of dietary assessment [45]. Uncertainties about registered diet records (ie, unclear handwriting, unclear food proportions) were solved by contacting the participant. Diet records were analyzed based on the nutrition values of the recorded products as shown in the Dutch nutrient database Nederlands Voedingsstoffenbestand version 2019/6.0 [46] and averaged over at least 2 available days.

##### Body Composition

Body mass was determined to the nearest 0.1 kilogram by deducting the mass of the wheelchair from the total mass of the participant and wheelchair combined, measured with a wheelchair weighing scale (RS1010, Allscales Europe used at Reade; Detecto 6550 used at Heliomare). Height and waist circumference (WC) were measured with a tape measure to the nearest 0.5 centimeters with the participants in supine position on a treatment table. WC was determined by taking the average of three measurements. BMI (body mass/height^2^) was calculated with the proposed equation of Himes [47] in the case of LLA, based on the relative body segmental mass determined by Osterkamp [48] to adjust for lost body mass. Fat mass and fat-free mass were measured with a Bodystat 1500MDD (used at Reade) bioelectrical impedance analysis (BIA) device or with a Bodystat 500 Touch (used at Heliomare) BIA device in supine position. The BIA electrodes were placed at the right side of the body after the participant was in supine position. In case of unilateral LLA on the right side, the left side was measured. Electrodes were attached on the hands and feet according to the user manual. The BIA formula of Kyle et al [49] was used to calculate the fat-free mass and fat mass percentages based on the measured reactance and resistance.

##### Physical Activity

Physical activity was measured by the Dutch Physical Activity Scale for Individuals with Physical Disabilities (PASIPD), a 12-item 7-day recall self-reported questionnaire evaluating physical activity level in individuals with a physical disability. The PASIPD outcome is the metabolic equivalents of task (MET) hours spent per day and was calculated according to the method of Washburn et al [50]. The score in MET (hours/day) can range from 0 to 182.3 and can differentiate significantly among various physical activity levels.

##### Self-Efficacy

The General Self-Efficacy Scale and Exercise Self-efficacy scale, which are valid and reliable 10-item questionnaires (4-point Likert scale, total scores range from 10 to 40, where a higher score represents a higher self-efficacy), were used to assess the general self-efficacy and coping ability in daily life and exercise self-efficacy [51-56].

##### Fatigue

The Checklist Individual Strength (CIS20R), a reliable self-reported questionnaire (20 questions answered on a 7-point Likert scale, total scores range from 20 to 140 where a higher score represents more severe fatigue), was used to measure multiple aspects of fatigue [56,57].

##### Sleep Quality

The Pittsburgh Sleep Quality Index (PSQI) is a valid self-reported questionnaire to evaluate overall sleep quality [58]. The questionnaire consists of 19 questions resulting in a global score between 0 and 21, which consists of seven component scores (ie, sleep quality, sleep onset latency, sleep duration, sleep efficiency, sleep disturbances, use of sleeping medication, and daytime dysfunction). A higher global score represents worse sleep quality. The PSQI is considered as a reliable and valid method to evaluate sleep quality [59].

##### Quality of Life

The short-form health survey enabled (SF-36E) questionnaire was constructed to measure health-related quality of life on eight different dimensions of health for individuals with a mobility impairment [60]. The eight dimensions are physical functioning, social functioning, physical role emotional role, mental health, vitality, bodily pain, general health, and health transition. Dimension scores are rescaled to a 0-100 score where a higher score represents a higher quality of life. The Dutch version of the SF-36E is considered as a reliable and valid tool for individuals with chronic disabilities [61].

#### Data Analysis

##### Qualitative Evaluation

The audio recordings were transcribed verbatim and analyzed using a content analysis approach [62]. After familiarizing with the data by listening to the interviews and reading the transcripts, initial codes were identified by labeling text segments (open coding). The open codes were a mix of inductive codes that arose from reading the transcripts and deductive codes that arose from the study aims and interview guide. The open codes were then organized in a thematic map by comparing them and categorizing them into codes and subcodes. Finally, the codes and subcodes were integrated into core categories or main themes, derived from the topics of the interview guide based on the SUS and USE questionnaires, and the broader literature on behavior change [40,41,43]. The coding was carried out by one researcher (JH), who discussed and agreed on the themes and codes with a second researcher (LtL). They discussed their findings with the research team to ensure reliability of coding and data interpretation. The transcripts were analyzed with MAXQDA version 11 (VERBI GmbH).

##### Quantitative Evaluation

All quantitative data were analyzed with IBM SPSS software (Version 26). Pre and postintervention changes were compared using a paired-sample *t* test. Normality assumptions were checked with the Kolmogorov-Smirnov test. If normality was violated, a Wilcoxon signed-rank test was performed. Significance was accepted at *P<*.05. Effect sizes were determined by Hedges *g*, except when assumptions were violated and the effect size was determined by z/(√n) [63]. Effect sizes can be interpreted based on the following: *g*<0.2 indicates a very small effect size, *g*=0.2-0.5 indicates a small effect size, *g*=0.5-0.8 indicates a medium effect size, and *g*>0.8 indicates a large effect size.

## Results

### Participants

**Figure 3:**
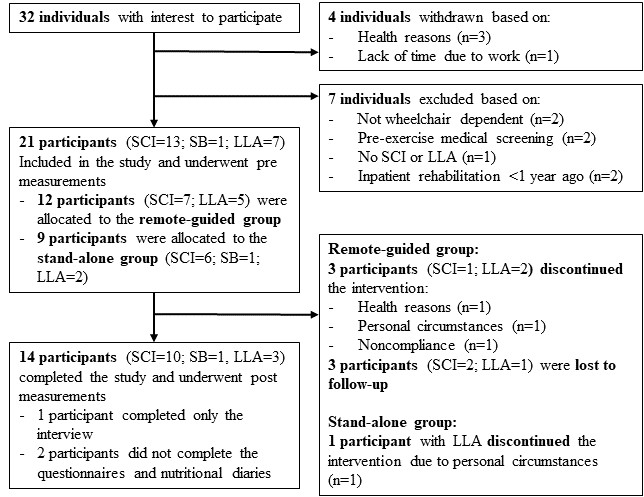
Flow chart of participant inclusion. LLA: lower limb amputation; SB: spina bifida; SCI: spinal cord injury.

Twenty-one participants were included in the process and effect evaluation study, 11 of whom completed all pre- and postmeasurements during the intervention period, as shown in Figure 3. One participant completed only the interview at the postmeasurement, and two participants did not complete the nutritional diaries and questionnaires at the postmeasurement. Demographic information of the participants who completed the intervention period are summarized in Table 1. No significant differences in demographic characteristics were found between the remote-guided and stand-alone groups. Because of the small group sizes, the results of the total population are presented. The results of statistical comparisons within the remote-guided and stand-alone groups are presented in Multimedia Appendix 3.

**Table 1:** Demographic characteristics of the participants.

Characteristics			Baseline (N=21)		Participants that completed the postintervention interview			
					Remote-guided (n=6)	Stand-alone (n=8)		Total group (n=14)
Gender (female/male), n			14/7		4/2	5/3		9/5
**Spinal cord injury, n**								
	Total	14		4		7	11	
	Tetraplegic	1		0		1	1	
	Paraplegic	13		4		6	10	
**Lower limb amputation, n**								
	Total	7		2		1	3	
	Unilateral	4		2		0	2	
	Bilateral	3		0		1	1	
Age (years), mean (SD)			51.6 (11.9)		55.5 (11.7)	54.1 (11.8)		54.7 (11.3)
Height (m), mean (SD)			1.68 (0.20)		1.74 (0.11)	1.64 (0.24)		1.69 (0.20)
Body mass (kg), mean (SD)			87.6 (21.5)		88.7 (19.8)	80.1 (19.4)		83.8 (19.3)
BMI (kg/m^2^), mean (SD)			33.7 (13.1)		30.0 (5.9)	31.6 (11.5)		30.9 (9.2)
Time since injury (years), mean (SD)			15.5 (15.8)		10.7 (6.3)	22.1 (22.5)		17.2 (17.8)

### Qualitative Evaluation

#### Prior Experience

Most participants (n=11) indicated they were reasonably to very experienced smartphone app users. Three participants did not consider themselves experienced with smartphones, including one participant who indicated that they mainly use a tablet, which was also used to run the WHEELS app. Six participants had no experience, two had minimal experience, and six had more extensive experience with lifestyle apps prior to participation in this study.

#### Themes

##### Overview

The codes and subcodes that emerged from the content analysis could be clearly classified under the dimensions of usability and feasibility of interest for this study. Therefore, the topics of the interview guide largely corresponded to the main themes used to categorize the results: (1) motivation and lifestyle goals, (2) app use and adherence, (3) satisfaction, (4) usefulness, (5) ease of learning, (6) ease of use, and (7) needs and suggestions for improvement. The themes were gathered and presented together for the app in general, and for the specific physical activity and exercise, food, sleep and relaxation, and community sections of the app.

##### Motivation and Lifestyle Goals

Motivations mentioned to participate in the pilot study were incentive to work on a healthy lifestyle, gaining insight into/becoming aware of physical activity and nutritional behavior, discovering new exercises, critically testing the app, and making suggestions for improvement. Participants mentioned at least one goal they hoped to achieve with support of the app. Ten of the 14 participants indicated weight loss as a lifestyle goal, 8 participants had goals related to increasing physical activity/exercise, 4 indicated healthy energy and/or food intake as lifestyle, 3 mentioned more overarching goals such as staying or becoming healthy and fit, and one participant indicated that he had a goal related to rest and relaxation, which was to fall asleep better.

Well, what I said: on the one hand to maintain fitness and also to maintain weight, because with age, weight goes on rather than it goes off. Especially when you sit all day, it is more difficult than when you walk, I think. So I would rather keep that stable and yes, in the positive case, I could also lose some weight.

Participant 5, female, 59 years old, LLA

Ten participants reported having partially achieved one or more lifestyle goals, five of whom indicated that they had lost weight. “And yes the food, I was very busy with losing weight. I succeeded, so it works well in that respect.” [Participant 9, female, 42 years old, SCI]

Different reasons were mentioned for why lifestyle goals were not (or only partially) achieved, such as bad weather (cold and wet); hay fever; personal circumstances, including the death of a loved one; laxity; stubbornness; shoulder injury; or having set too ambitious goals. Additionally, the goal of improving falling asleep was difficult to target specifically. *“*I wanted to lose 5 kilos and in the period I was working on this I already realized that this was a bridge too far.” [Participant 4, male, 67 years old, SCI]

Partially achieving the lifestyle goals was not always entirely attributed to use of the app. Some participants indicated that participating in the pilot study, and therefore consciously working on a healthy lifestyle, already provided sufficient incentive to pursue existing lifestyle goals. Nevertheless, the majority indicated that the app had influenced their physical activity and dietary behavior, particularly contributing to raising awareness of, and providing insight into, daily energy and nutrient intake.

Because normally, when there are some things on the table you really have no idea how many calories are in it. And if you look a little further in the app, you can also see how many carbohydrates and other macronutrients are in it. So it was firstly to build up a bit of discipline: what will I shove in during the day. And secondary, a piece of awareness was built up. Then you have eaten a nice pizza, and suddenly get 1200 calories on your plate, and then you are not allowed to eat anything for the rest of the day.

Participant 1, male, 64 years old, SCI

In addition, the participants explained that the app provided incentive discipline to exercise regularly and to eat healthy, with pop-up reminders to exercise contributing to this.

I thought I should honestly fill in what I eat and drink and then you see your own overview… Then [when the overview shows a surplus in calories] you think: another day tomorrow, I have to fix this right away.

Participant 17, female, 57 years old, SCI

I definitely do less now without the app. Because the app sort of said: now it's time for your weekly gym exercise.

Participant 19, male, 39 years old, SCI

Finally, it was mentioned that the app provided direction and tools to adopt a healthy lifestyle, such as by offering exercises.

Look, I sit in a wheelchair and I don't do anything else. But now you see oh, I can do that and I can do that and I can do that… And now I have come this far, also together with my physiotherapist, that I get out of that wheelchair. That I look at what kind of standing exercises and suchlike I could do, and that motivates me enormously.

Participant 16, female, 70 years old, LLA

##### App Use and Adherence

Six participants reported having used the app daily, two had used the app extensively during the 12-week intervention period, but not daily, whereas five had used the app only in the beginning (2-5 weeks), and one had used the app irregularly. Three reasons were given by several participants for having used (certain parts of) the app less or not at all. The first reason was dissatisfaction with the functioning or ease of use of the app: “If at some point you feel that it is not working in the way you would like, yes, then you think forget it.” [Participant 1]

The second reason was personal circumstances such as an injury, illness, and psychological stress: “I have also seen the cardio program. There was something about building up biking, but just because I was not in good shape, I didn't start doing that.” [Participant 2, female, 64 years old, SCI]

The third reason given was laziness:

I did not use that [food part]

Interviewer: *Okay and what was the reason for that?*

Yes, laxity, ease. Maybe also the stubbornness that you think: yes, I know how to lose weight.

Participant 11, male, 61 years old, SCI

The food diary was used most intensively, which was completed almost daily by six participants. Four others also used the food part but less intensively or quit after a few weeks. Another participant only tested the food part for usability.

Four participants used the exercise database and preprogrammed exercise routines daily or frequently during the 12 weeks. One participant also used these parts of the app regularly, although less frequently. Four participants used the physical activity and exercise part in the beginning but stopped after a few weeks. In addition, one participant had only explored the exercise database and training programs and another participant only tested the physical activity and exercise part for usability. Competitive activities were mentioned as one of the reasons for no or little use of the exercise part: “In the beginning I also used the app, but because I went to the gym twice a week, I stopped using it.” [Participant 2]

A second reason given to stop using the exercise part was that the app was no longer needed because the exercises were known and could be performed without the app.

Yes, those were just example exercises and then I thought: oh yes, that is a fun one, oh I am going to do that, and: oh, that is also a fun one that I am going to do and then you have four or five [exercises]. Yes, then I really don't need that app anymore. Because then I already know what to do. Then I no longer have to look at that app every day.

Participant 5

Three participants had used the sleep and relaxation part. The first participant performed the relaxation exercises several times, the second had read the information about sleep and relaxation, and the third explained that he still used the tips to relax because he slept rather poorly and the tips helped him to relax. A fourth participant only tested the sleep and relaxation part for usability. A frequently mentioned reason for why the sleep and relaxation part had not been used was that it was not needed because participants did not experience stress or sleeping problems: “Not looked at [sleep and relaxation part] because I am sufficiently balanced and relaxed.” [Participant 11]

Three participants used the informative community groups. They were alerted by email to new messages in which lifestyle information and tips were shared and read them. The interactive part of the community in which participants could ask each other questions, and share experiences and tips was hardly used. One participant expressed that it was unfortunate that hardly any interaction had started. Reasons given for not being active in the community were unwillingness to brag and incomparability.

It is more because it is so incomparable to each other. Look, someone who just got out of rehabilitation may be very proud to have handcycled 10 kilometers. While I think, yes, when I say I have done 20 kilometers... I think that...I don't feel the need to proclaim it or anything. I think, yes, you are not comparable.

Participant 5

Finally, the calendar was used by several participants to plan their exercises. Two participants reported having used the progress registration part by regularly registering their weight and WC. A few participants started the handcycling challenge; however, no participant completed this challenge because it was unclear how the time that was handcycled for this challenge could be recorded.

##### Satisfaction

Overall, participants were satisfied with the WHEELS app. Several participants indicated that they wanted to continue using the app. It was further noted they were happy that there is finally a lifestyle app suitable for wheelchair users, that the combination of attention to healthy nutrition and physical activity/exercise is nice, and that the app has something in it for everyone.

I think that because you have a lot of different things in it, you have a very large target group. Some things may not be helpful to me, but that's not to say it's not useful for the app.

Participant 9

When asked if they would recommend the app to others, 12 participants answered yes, one participant would only recommend the food part, and one person would not recommend the app at all: “Maybe, I haven't thought about it. But there is, yeah, it's a good way to start up. Until you get into a routine.” [Participant 19]

With regard to the exercise database and preprogrammed exercise routines, the participants indicated that there was great variation in exercises and that the animation provided a clear example of the desired implementation: “I have to tell you, I think it looks super cool. Also the exercises that are offered, I think the variation in exercises is very good.” [Participant 11]

Some participants were less satisfied with the exercise database because they had difficulty finding the right exercises: “And then you see so many exercises in that list that you actually do not know which one to choose. And that was a problem for me.” [Participant 7, male, 54 years old, SCI]

Regarding the food part, the participants indicated that they liked that that the app provided insight into their daily energy and nutrient intake. Furthermore, they explained that the food product list was very extensive, as almost every food could be found in the list and then easily added to the food diary.

I found the food diary very useful, you can see exactly what you eat in calories and protein.

Participant 8, female, 34 years old, SCI

The nice thing about the food app is that no matter what you eat or drink, you can always find it somewhere and it has a calorie number.

Participant 1

The relaxation part was rarely used, making it difficult to indicate whether the participants were satisfied with the content. Two participants indicated that the exercises were perhaps a bit too spiritual: “Well it often comes across as very spiritual, so to speak, while, yes, while that might raise an aversion.” [Participant 9]

With regard to the community aspect, a few participants stated that it was motivating that lifestyle tips were regularly posted. Opinions were divided about the community group in which experiences and questions could be shared: some considered this to be of added value, whereas others did not feel the need for it.

##### Usefulness

The participants indicated that the app is particularly useful for (recent) wheelchair users who have little physical activity and exercise experience, providing tools and inspiration to start exercising and develop a healthy lifestyle.

Because I see a lot of wheelchair users around me who are simply aimless. Who can have a huge hold on that [the app]. Especially if you are not physically active or are starting to be physically active.

Participant 11

More experienced wheelchair users with a more physically active lifestyle had less need for the complete app, but found some parts useful, in most cases concerning the food part to gain insight into dietary intake, as described in the previous section on satisfaction.

##### Ease of Learning

Opinions differed on how easy it was to learn how to use the app. Some participants quickly became skillful, others needed a few weeks, and still others gave up using the exercise part because they could not learn to work with it quickly. Most interviews showed that learning to work with the app takes some time, and not all participants had the patience and will to spend time on this.

The first period is quite a lot of investing and maybe I could have got a bit more out of it. But then you expect all users to use it, invest and maybe benefit from it afterward, and I think that is too much to ask.

Participant 17

Some participants indicated they could not repeat actions they had previously performed with the app, such as scheduling an exercise in the calendar. This indicates it was not easy for all participants to remember how to use the app. Some participants also indicated they had asked family members to help them learn to work with the app. This also reveals that learning to work with the app was not experienced as easy by all participants.

Actually during the first 2 or 3 weeks of use, you don't know all the tips at once, because that is too much, but then the advantage is that my daughter is very handy and serious with that [app use]. So then I got another tip and I could do a bit more. Yes, perfect that app.”

Participant 12, female, 61 years old, LLA

The part that participants most often reported as unsuccessful or difficult to master was putting together a training schedule themselves.

Well, what I said earlier about making such a [exercise] program of your own and then...Yes that. I could not completely figure that out, and I must honestly say that at some point I also think: well, never mind.

Participant 5

Finally, the participants hardly reported any problems with learning to work with the food part. This part seemed to work quite intuitively.

##### Ease of Use

The participants generally found the food part easier to use than the exercise part. The vast majority labeled the food part as easy to use, whereas the exercise part was described as easy to use by about half of the participants.

Three factors emerged that negatively affected the ease of use of the exercise part. Not all participants were well aware of the distinction and coherence between the exercise database with which personal training routines could be compiled, the preprogrammed exercise routines, and the calendar.

Well with those exercise programs I found it a bit vague at first because you also have two boxes [tiles at the start screen that direct to the different parts of the app] with exercise or something.

Participant 8

Second, the participants indicated they had difficulty finding suitable exercises in the exercise database. Several participants were unaware of the possibility or did not know how to use the search box to find specific exercises.

For example, if I had done something new at the physio, it was sometimes a bit of a search in that whole list of: What suits this best? And then indeed there were sometimes whole laundry lists with exercises in it...I think that at some point it just missed its target...[Participant 4]

Third, it was not clear to all participants how activities could be registered to end up in the activity diary or the activity stream.

And for example I had put an exercise in the calendar and then I thought oh then it will automatically register that I did that, but that is not the case. Then you had to click that again.

Participant 8

The participants generally found the food part clear to use. Several participants indicated that they liked the fact that the food product list was so extensive that it was easy to log consumed products in the food diary: “Yes, I actually did not come across a product that I could not find [in the food product list].” [Participant 14]

The participants found the time it took to keep a food diary less user-friendly. Several participants were not aware of the possibility to save frequently eaten meals such as a fixed breakfast. This means that the individual products that make up the meal only need to be entered once. The next time, the entire meal can be added to the food diary with one click. Partly because participants were not aware of this possibility, they indicated that they were tired of having to import the same products every time.

At some point you are a bit done with reentering those foods every time. I always eat a bowl of curd with muesli in the morning. Yes, the next day I had to scan it again or had to go back and find out where that curd is. And I thought that was a bit of a disadvantage.

Participant 7

##### Needs and Suggestions for Improvement

The participants expressed several needs and suggestions for improvement that were largely related to the difficulties they experienced during app use. The most frequently mentioned needs and suggestions related to personalization, user instruction or remote guidance, and improving insight into energy expenditure by connecting a wrist-worn activity monitor to the app. No difference in suggestions was found between the remote-guided and stand-alone groups.

In the exercise part, better personalization could make it easier to find and select suitable exercises. In addition, it was suggested that the preprogrammed exercise routines could be better tailored to the individual needs and functional capabilities: “However, I would appreciate the programs to be a little bit more distinct to your abilities. That would have been perfect.” [Participant 19]

With regard to the food part, the participants expressed their need for personalized daily energy intake advice: “If you want to use it for people with a spinal cord injury, you really have to adjust the calorie advice, because we are actually allowed to consume fewer calories.” [Participant 7]

The user instruction was not found and used by all participants. Some participants indicated they needed a help desk for questions and problems with using the app.

That I couldn't find back what I had done before was the most frustrating...That I didn't know how I had done it before and why I couldn't find it again…Someone who offered me guidance, by phone or every 2 weeks face-to-face, could have definitely helped me.

Participant 17

In addition, several participants would have liked to have had a coach to help them on their way, have questions answered, and discuss suitable activities and progress on their goals. One of the participants who had received remote guidance explained that it had been of little added value to her because the coach barely reflected on what she had done. She would have liked the coach to monitor her and to discuss progress on her goals with her: “Yes someone who says: you went over your [calorie] goal all week. Do you have an idea how you will solve this in the coming week?” [Participant 14, female, 42 years old, SCI]

Finally, it was revealed that, in combination with lifestyle guidance by a coach, the app would probably have been used more intensively: “I think I would have used it under supervision more, the WHEELS app.” [Participant 17]

As a third and last suggestion, several participants explained that they would like to have better insight into their rate of exertion and energy expenditure during activities. Connecting an activity monitor for wheelchair users to the app has been suggested as an opportunity to meet this need.

Yes, a pedometer too, for a wheelchair user. It is nice to know what your muscles have done, what your heart rate has been and how many calories or energy you have consumed…I would rather put on a wristband for that and will also enter some extra information.

Participant 17

### Quantitative Evaluation

#### Usability and Feasibility

The average SUS score for all participants was 58.6, which is below the general average interface SUS score of 70 [44]. Participants showed little difference in scores between the remote-guided and stand-alone groups in SUS score and USE dimension scores. All SUS scores and USE dimensions scores can be found in Table 2 for each group and all participants.

**Table 2:** Descriptive statistics of usability and feasibility questionnaires.

Questionnaire	Remote-guided (n=4)		Stand-alone (n=7)		All (N=11)	
	Mean (SD)	Range^a^	Mean (SD)	Range	Mean (SD)	Range
System Usability Scale	56.0 (5.8)	14.0	60.1 (32.2)	84.0	58.6 (25.2)	84.0
Ease of use	5.0 (1.7)	4.3	5.6 (3.8)	9.7	5.4 (3.1)	9.7
Satisfaction	5.1 (2.2)	4.8	5.2 (3.7)	9.7	5.2 (3.1)	9.7
Ease of learning	5.5 (3.2)	7.0	6.1 (4.2)	9.5	5.9 (3.7)	9.5

^a^Represents the spread; the difference between the maximum and minimum values.

#### Nutritional Intake

Nutritional intake changes of 10 participants over the 12-week intervention period are shown in Table 3. The quality of the diet records of two participants was not adequate (ie, portion size or ingredients used were not described in sufficient detail to obtain macronutrients correctly) to analyze at least 2 recorded days at pre and postmeasurement, and these records were therefore excluded. No significant differences were found over time within the whole group, or within the remote-guided or stand-alone groups separately, in total calorie count or macronutrients. However, there was a positive trend (with medium effect sizes) toward a reduction in fat consumed and relative alcohol intake. Other outcomes showed either small or very small effect sizes. Full results of the separate groups are presented in Table S1 in Multimedia Appendix 3.

**Table 3:** Nutritional intake pre and post the 12-week intervention based on diet records (n=10).

Daily average consumed	Pre, mean (SD)	Post, mean (SD)	*P* value	Effect size
Kilocalories	1920 (531)	1637 (377)	.14^a^	0.46
Protein (g)	79.8 (21.8)	74.1 (13.5)	.37	0.28
Fat (g)	82.9 (31.4)	69.6 (22.9)	.07^a^	0.56
Carbohydrates (g)	179.8 (61.3)	154.7 (63.3)	.24^a^	0.37
Alcohol (g)	12.7 (13.3)	7.9 (10.7)	.16^a^	0.44
Protein (%)	16.9 (3.7)	18.6 (3.6)	.14^a^	0.47
Fat (%)	38.4 (8.5)	38.2 (8.6)	.88	0.05
Carbohydrates (%)	37.7 (8.1)	37.3 (10.3)	.88^a^	0.05
Alcohol (%)	4.5 (4.7)	3.4 (5.0)	.09^a^	0.53

^a^Nonparametric test used due to violation of normality with corresponding effect size.

#### Body Composition

For the whole group, all body composition outcomes showed favorable changes over the intervention period, which was significant for WC, fat mass percent, fat-free mass, and fat-free mass percent, and a trend toward significance for body mass and BMI (Table 4). Large effect sizes were found for fat mass percent and fat-free mass percent, whereas all other outcomes showed medium effect sizes except for body mass. Results for each group separately are shown in Table S2 in Multimedia Appendix 3, demonstrating slightly better results in favor of the remote-guided group compared to the stand-alone group.

**Table 4:** Body composition changes pre and post the 12-week intervention (n=13).

Body composition	Pre, mean (SD)	Post, mean (SD)	*P* value^a^	Effect size
Body mass (kg)	83.0 (20.0)	81.6 (20.6)	.09	0.49
BMI (kg/m^2^)	29.7 (7.8)	29.1 (7.8)	.07	0.53
WC^b^ (cm)	103.9 (13.5)	101.4 (13.8)	.02	0.76
FM^c^ (kg)	32.1 (10.1)	26.7 (9.2)	.004	0.96
FM (%)	39.2 (9.5)	33.4 (10.5)	.004	0.97
FFM^d^ (kg)	50.9 (15.4)	54.9 (17.7)	.02	0.70
FFM (%)	60.8 (9.5)	66.6 (10.5)	.004	0.97

^a^Paired-sample *t* test.

^b^WC: waist circumference.

^c^FM: fat mass.

^d^FFM: fat-free mass.

#### Questionnaire Outcomes

No significant changes in questionnaire outcomes were found over time (Table 5), although the PSQI results showed a favorable trend toward better sleep quality. A medium effect size was found in sleep quality. Small effect sizes were found on the CIS20R and four subcategories of the SF-36E. No clear differences were found between groups, as shown in Table S3 in Multimedia Appendix 3.

**Table 5:** Results from questionnaires pre and post the 12-week intervention (n=12).

Questionnaire		Pre, mean (SD)	Post, mean (SD)	*P* value	Effect size
PASIPD^a^ (MET h/day)		23.8 (15.1)	22.4 (12.2)	.64	0.14
GSES^b^		34.1 (4.3)	33.6 (4.2)	.69	0.11
ESES^c^		33.0 (3.8)	32.4 (4.4)	.50^d^	0.07
CIS20R^e^		72.3 (11.6)	69.6 (7.7)	.18^d^	0.39
PSQI^f^		8.0 (2.7)	6.7 (2.2)	.06	0.57
**SF-36E^g^**					
	Physical functioning	52.1 (18.6)	49.2 (18.2)	.58	0.16
	Social functioning	72.9 (17.5)	79.2 (20.9)	.36^d^	0.27
	Role limitation physical	57.8 (26.1)	59.4 (18.0)	.84	0.06
	Role limitation emotional	79.2 (23.7)	74.3 (24.7)	.53^d^	0.18
	Mental health	81.7 (12.6)	74.6 (15.1)	.13	0.46
	Energy/vitality	62.5 (14.6)	62.5 (12.2)	>.99	1.00
	Pain	59.0 (17.6)	49.1 (21.5)	.11	0.48
	General health perceptions	63.8 (18.1)	60.0 (21.3)	.37^d^	0.26

^a^PASIPD: Physical Activity Scale for individuals with Physical Disability.

^b^GSES: General Self-Efficacy Scale.

^c^ESES: Exercise Self-Efficacy Score.

^d^Nonparametric test used due to violation of normality.

^e^CIS20R: Checklist Individual Strength.

^f^PSQI: Pittsburgh Sleep Quality Index.

^g^SF-36E: Short-Form Health Survey 36 Enabled.

## Discussion

### Principal Findings

This paper describes the development of the WHEELS mHealth app for wheelchair users with SCI or LLA. Additionally, the first insight on usability, feasibility, and effectiveness of the app is provided. The perceived usability and feasibility varied among participants and showed room for improvement. Participants did show a positive development in body composition such as a significant decrease in fat mass, which was often mentioned as a personal lifestyle goal. Combined with a positive trend for sleep quality, and reduced fat and alcohol intake, the app seems promising to improve lifestyle behavior in wheelchair users, with the caveat that no change in physical activity levels were detected. Environmental barriers might have contributed to this, which cannot be influenced by mHealth.

The SUS score and usability scores for ease of use, ease of learning, and satisfaction ranged between the minimal (0) and maximal (10) scores, indicating a varied user experience. The questionnaire outcomes were in line with the interviews, in which some participants were merely positive and experienced no struggle using the app, whereas others mentioned difficulties using the exercise and planning part, for example. These differences in perceived usability could be related to differences in motivation and time spent within the app, and likely influenced the extent to which the app has led to the desired lifestyle behaviors. This is best explained by the Fogg behavior model, which describes that behavior change is related to three elements, motivation, ability, and trigger, where motivation and ability show an inverse relationship [64]. If a certain level of effort and time (motivation) was not put into understanding the app (ability), an individual would not meet the minimal requirements to benefit from the app. Participants who were willing to put more time and effort in becoming familiar with the app, and thus showed more motivation, were more positive about the product and expressed fewer difficulties working with the exercise and planning part. This is in line with earlier research, which shows that a higher level of app engagement is associated with increased intervention effectiveness [65-67]. However, this could also be a flaw of the app, as the required ability might be too high to fully benefit from the app. Therefore, by reducing the ability needed to understand the app, less motivation is needed to continue using the app. Clearer and easier instructions that require little time and effort could possibly reduce the required motivation to meet the ability needed to benefit working with the app.

Another solution for overcoming difficulties with using the app could be found in a remote-guided intervention approach, in which the app is combined with guidance by a lifestyle coach. A remote-guided approach seems to be more promising in achieving improvement in behavioral and health outcomes [25]. Unfortunately, from this study, no conclusions can be drawn regarding differences between those who used the app as a stand-alone intervention and those who used the app with remote guidance. The remote-guided group did show more significant changes than the stand-alone group; however, owing to a larger dropout than expected, the group sizes were small and thus results should be interpreted with caution. Half of the participants were allocated to a remote-guidance group where they received regular phone consultations by students in Functional Exercise Therapy. However, based on interview reports, the effect of the provided phone consultations was limited, possibly due to the lack of experience the students had in motivational interviewing. Previous research suggests more advanced and prolonged training in motivational interviewing is needed to allow embedding of these skills [68]. Another explanation for the limited effect is that nonverbal communication was hardly possible because most consultations took place by phone, which could have reduced the consultation effects due to loss of possible valuable cues and information [69]. Moreover, multiple participants from the stand-alone group indicated that a consultant would have benefitted them in either solving difficulties in working with the app or as an additional motivator and guide in behavior change. The addition of peer health coaches could be of added value, as research shows that individuals with SCI can benefit from this type of support [70]. Therefore, it would be interesting to investigate the effect of using the app in combination with face-to-face guidance of trained peer health coaches (blended).

When taking a closer look at the effect evaluation, significant and favorable changes were seen in measures of body composition. This seems to be in line with other findings such as reduced body mass, reduced fat intake, and reduced relative alcohol intake, although these reductions were not statistically significant. Registered body composition changes were most likely partly caused by nutritional changes. The feature to track nutrition intake raised the participants’ awareness of their nutritional intake and triggered them to change dietary habits, which was also mentioned during the interviews. The diet records showed a trend toward a positive change in nutrition behavior. These changes were not statistically significant, possibly due to the small sample size. No changes in physical activity levels were found, which could be caused by factors at the interpersonal, institutional, community, and policy influence levels that were not targeted in the app but are associated with physical activity among wheelchair users [71]. However, the significant increase in fat-free mass would suggest an increase in muscle mass caused by physical activity. Moreover, physical activity was measured with a self-reported questionnaire, which correlates poorly with objective physical activity outcomes in participants who were already relatively active at the start of the study [72]. Therefore, there may have been an increase in physical training that was not reflected in the total PASIPD score.

### Limitations and Strengths

The targeted groups, wheelchair users with SCI and LLA, may experience different barriers and facilitators for developing a healthy lifestyle, and when developing the app we expected that the app had to be tailored accordingly. However, the needs assessment showed many similarities, resulting in the use of similar behavior change techniques for both groups. The intention was to use the 16 change strategies in the intervention to influence the main behavioral determinants identified in the development phase. However, it is uncertain whether all 16 strategies were applied as intended during the study. For example, tailoring options were limited due to software limitations, and several participants had not used all parts of the app with the result that they were not exposed to all behavior change strategies. This could have possibly affected the usability and effectiveness outcomes.

The interview yielded suggestions for improvement that could in turn improve the usability, feasibility, and effectiveness of the app. However, owing to high dropout, relevant feedback may have been missed from users who were less satisfied with the intervention or had more difficulty changing their behavior. Unfortunately, in most cases, we were unable to determine the reason for dropout, as this could have provided valuable information. The relative high dropout rate led to a lower sample size than intended and a possible biased user experience. Additionally, this resulted in only one individual with tetraplegia completing the study, making these results less generalizable to the whole SCI community. Nevertheless, despite the low inclusion rate, significant positive changes in body composition were found, which is very promising. However, these results should be interpreted with caution, as a higher possibility of type II errors is present due to the small sample size and multiple performed tests.

Several body composition outcomes were measured with a BIA device. The transformation formula used to calculate measured resistance to body composition outcomes was based on empirical data from the general population. Thus, the validity of the BIA measurements on this specific population could be argued. However, systematic deviation does not have to affect test-retest reliability, which would therefore make the BIA still able to detect changes over time. Additionally, the BIA outcomes seem to be in line with interview outcomes and nutrition diaries. Physical activity levels did not show any changes, which was subjectively measured with the PASIPD questionnaire and, as mentioned above, correlates poorly with objective physical activity outcomes that represent physical activity more accurately [72].

### Future Studies

These first results on effectiveness of the WHEELS app seem to be promising for body composition changes, nutritional habits, and sleep quality. Improvements in manual instructions and support regarding use of the app are suggested. A study with a larger sample size and stronger research design, for example a repeated-measures mixed model design, is warranted, which would allow further investigation on the effectiveness of the different parts of the app for improving body composition, dietary behavior, physical activity, and health, and the interaction between stand-alone and remote-guided use of the intervention. In this larger study, it is recommended to measure physical activity objectively (eg, with accelerometry) to be able to conclude whether the app does or does not influence physical activity behavior. Preferably, such a study should be performed in participants who are less active at inclusion compared to the participants of this study. Accelerometry, including heart rate, would be recommended, as it could differentiate among intensity levels and thus provide a more valid physical activity outcome.

### Conclusion

This paper describes the development, usability, and feasibility of the WHEELS mHealth app for wheelchair users with SCI or LLA, and provides the first insight into its effectiveness. Although usability could be improved, the app scored reasonably well and seems to be feasible to implement on a larger scale. First results on lifestyle changes seem promising, and effectiveness could possibly increase if the mentioned suggestions for improvement by participants are processed into the app.

## Appendix

Multimedia Appendix 1 Development process of the WHEELS app.

Multimedia Appendix 2 Interview guide for the usability and feasibility study of the WHEELS project.

Multimedia Appendix 3 Tables S1-S3.

## Abbreviations

BIA: bioelectrical impedance analysis
CIS20R: Checklist Individual Strength
LLA: lower limb amputation
MET: metabolic equivalents of task
mHealth: mobile health
PASIPD: Physical Activity Scale for Individuals with Physical Disabilities
PSQI: Pittsburgh Sleep Quality Index
SCI: spinal cord injury
SF-36E: Short-Form Health Survey Enabled
SUS: System Usability Scale
USE: Usefulness, Satisfaction, and Ease of use
WC: waist circumference
WHEELS: Wheelchair ExercisE and Lifestyle Study
