# Supplementary material for: Mobile App (WHEELS) to Promote a Healthy Lifestyle in Wheelchair Users With Spinal Cord Injury or Lower Limb Amputation: Usability and Feasibility Study
Source: JMIR Form Res. 2021 Aug 9;5(8):e24909. doi: 10.2196/24909 (PMC8386360; doi:10.2196/24909)
Supplement: Multimedia Appendix 1 [file formative_v5i8e24909_app1.docx]

## Multimedia appendix 1 Development process of the WHEELS app

### Step 1: needs assessment and intervention goals

At the start of the project a planning group was established to help develop the intervention. This planning group consisted of a representative of the target group (i.e., board member of the Dutch SCI patient association), rehabilitation professionals (i.e., exercise therapists and occupational therapists) working at two Dutch rehabilitation centers (Reade, Amsterdam and Heliomare, Wijk aan Zee), human movement scientists, health scientists, eHealth experts and a software development manager. Subsequently, the lifestyle behaviors – in terms of physical activity, nutrition and relaxation/sleep – and related problems in health and health-related quality of life among persons with SCI or LLA, were investigated through a literature search and focus group interviews. In addition, determinants of these lifestyle behaviors and contributing environmental conditions were identified. To analyze all data the PRECEDE model was used [1].
It has been shown that the PA level (measured by accelerometry) of wheelchair users with SCI and wheelchair users with LLA caused by vascular diseases, is less than 40% of the able-bodied level [2,3]. In 2017, only 12% of Dutch people with a motor impairment aged 12 years and older, including persons with SCI or LLA, met the Dutch physical activity guidelines and 21% practiced sports weekly versus, respectively, 46% and 54% of the same age group in the general population [4,5]. Prolonged sedentary behavior is common in wheelchair users and considered as a modifiable risk factor for cardiovascular diseases [6]. New SCI PA guidelines were published in 2017 including an infographic, focused on wheelchair users [7,8]. Because amputation PA guidelines are not specified for wheelchair users, the SCI PA guidelines were also applied for the LLA individuals as intervention goal, as this guideline is more applicable to persons for whom prolonged sitting is unavoidable. In addition, it has been shown that persons with SCI and LLA have a poorer diet quality compared to the general population, characterized by inadequate intake of dairy, fruit, whole grain foods and fibre, and a too high intake of fat, sugar and sodium [9–11]. Furthermore, sleep disturbances and psychological distress have been reported in both groups, and fatigue is known to negatively affect the lives of persons with SCI [12–18].These unhealthy lifestyle behaviors and lack of vitality are directly, or via being overweight, linked to an increased risk of physical and psychological comorbidity [11,19,20] and a reduced quality of life [21,22].

These findings from the literature were confirmed by a total of seven focus groups. Five focus groups with wheelchair users (n=25) with an average age of 58 years ranging between 39 and 75 years took place. Most included participants were fully adapted to their chronic condition with an average time of 9 years with a range between 0 and 55 years since the onset of their chronic condition. Two focus groups with rehabilitation professionals (n=11), with an average of 10 years of working experience ranging between one and 30 years, were held to gain a clearer picture of the needs of wheelchair users regarding lifestyle guidance [23]. Based on this needs assessment the following intervention goals were determined.

1. Wheelchair users with SCI or LLA comply with the scientific exercise guidelines for adults with SCI [7].
2. Wheelchair users with SCI or LLA have a healthy energy balance;
3. Wheelchair users with SCI or LLA have a healthy balance between exercise and sleep/relaxation; and
4. After the rehabilitation phase, rehabilitation professionals offer lifestyle guidance to wheelchair users with SCI or LLA with support of the application.

### Step 2: matrices of change objectives

In this step, the desired health promoting behaviors were specified. First, the desired behaviors of wheelchair users (intervention goals 1–3) and rehabilitation professionals (intervention goal 4), were broken down into subcomponents or performance objectives by answering the question: “What does the wheelchair user or professional need to do to attain the desired behavior?”. Subsequently, a stakeholder group (n=19) – consisting of wheelchair users with either SCI or LLA, partners of wheelchair users, rehabilitation professionals, health scientists and human movement scientists – was asked to rate the importance of achieving these performance objectives for developing the desired health promoting behaviors on a seven-point rating scale ranging from “not important” to “very important”. Based on this stakeholder analysis twelve performance objectives for physical activity and exercise behavior, seven for dietary behavior, five for sleep and relaxation behavior and four for the behavior of rehabilitation professionals were selected to further use in developing the intervention.

Secondly, modifiable behavioral determinants that should be targeted with the intervention were selected based on the results from the aforementioned focus group study [23,24], a literature search, a stakeholder analysis and theory. In the stakeholder analysis, 23 stakeholders – largely the same persons who ranked the performance objectives – rated the importance of a large number of determinants for developing and/or maintaining healthy physical activity, dietary and relaxation behavior on a seven-point rating scale ranging from “not important” to “very important”. In the end, six modifiable determinants were selected to target in wheelchair users, i.e., attitude [25], awareness, self-efficacy [26], knowledge [27], outcome expectations [28,29] and skills [30]. To target in rehabilitation professionals, the determinants perceived barriers, knowledge, social support and skills were selected.

Finally, matrices of change objectives were created by combining the performance objectives with the determinants. Change objectives answer the question what needs to change in the determinant for the wheelchair user or rehabilitation professional to achieve the performance objective. These matrices are provided in the Matrices of change objectives created in the WHEELS project to direct the development of a mobile lifestyle intervention for wheelchair users).

### Step 3: behavior change methods and practical applications

After creating the matrices of change objectives, theory- and evidence-based methods for achieving the intervention goals were chosen based on the determinants selected in step 2. These methods were then translated into applications features, i.e. specific strategies to deliver the method in a way that suits both the wheelchair users and rehabilitations professionals and the intended eHealth setting [31]. The behavior change methods and matching practical strategies were derived from various books and scientific articles, the focus group results and a stakeholder survey [32–40]. In this stakeholder survey an initial list of practical strategies was presented to 19 stakeholders with the question to rate the importance of each strategy for developing and/or maintaining healthy physical activity, dietary and relaxation behavior on a seven-point rating scale ranging from “not important” to “very important”. The selection process yielded 16 methods – i.e., tailoring, self-monitoring, providing feedback, modeling, facilitation, direct experience, persuasive communication, active learning, consciousness raising, personalizing risks and benefits, goal setting, setting graded tasks, planning coping responses, motivational interviewing, guided practice and participation – which were mainly derived from the social cognitive theory, the elaboration likelihood model and transtheoretical model of change [32]. An overview of the determinants, their linked behavior change methods and practical strategies is provided in appendix 3 (Multimedia appendix 3: Behavior change methods and practical strategies used to achieve the intervention goals of the WHEELS project).

### Step 4: pretesting, program refinement and production

In step 4, the information collected in IM-steps 1 to 3 was used to develop intervention materials and the mobile lifestyle app. First, six personas were created to ensure the lifestyle app would match the needs and potential of a heterogeneous group of future users. In order to create heterogeneous personas, variation was applied on the following aspects in order to ensure heterogeneity in the different personas: age, sex, educational level, severity chronic condition, physical function, mental state and social situation. A wide range in age was applied 26 – 75 with a 4/2 male/female ratio as it would represent Dutch society most regarding the presence of SCI and LLA. Severity and therefore physical function ranged in case of SCI between paraplegia and tetraplegia with complete or incomplete lesion. In case of LLA, severity ranged between unilateral transtibial amputation and bilateral amputation. Three personas were created with an SCI and three with LLA. Personas were constructed in such a way that the combination of characteristics of these aspects would be very feasible and would represent a potential future user of the app. Unlikely combination were avoided, such as: elderly with young children. Three rehabilitation professionals reviewed the personas and made suggestions for improvement to ensure they were a true reflection of the wheelchair users they encounter in their daily practice. Second, two independent eHealth experts linked the list of practical strategies to design principles for persuasive system content and functionality based on the Persuasive Systems Design model [41]. They translated the list of practical strategies and linked design principles into user requirements and checked whether these met the needs of the personas. Finally, based on the list of practical strategies and user requirements, the app was built by the Dutch software provider Virtuagym using their already existing software solutions for the fitness and health industry.

Figure 1 shows the structure of the WHEELS-app. The tiles on the home screen direct the user to the different parts. The “Community” includes a start instruction and four groups that provide information on: 1) physical activity and exercise; 2) healthy eating and energy expenditure; 3) sleep and relaxation; and 4) lifestyle change tools, such as creating an action plan, coping plan and payoff matrix. A fifth group allows users to ask questions, share experiences and tips, and interact with each other. The “Individual exercises” and “Exercise program” tiles direct to an exercise database with more than 250 custom-built exercises suitable for wheelchair users and pre-programmed exercise routines. In the “Food” part users can keep a food diary and get insight into their daily energy and nutrient intake. In the “Sleep & Relaxation” environment relaxation exercises are offered, in addition to knowledge transfer about balancing physical and mental load and capacity, healthy sleeping and relaxation habits. Behind the “Progress” tile users can get insight into changes in predefined health and fitness parameters, such as weight and body mass index (BMI). In addition, the app offers the ability to take on various lifestyle challenges. For example, the user can participate in the 90-minute weekly handcycling challenge and compare his/her performance with that of other participants. Finally, the app contains a calendar in which exercises and exercise routines can be scheduled.

The information and relaxation exercises provided in the community have been developed by the project group in collaboration with fourth-year bachelor students Sport studies, Functional Exercise Therapy and Nutrition and Dietetics who conducted literature research and interviewed rehabilitation professionals with expertise in exercise, nutrition and relaxation/sleep. The information on exercise and physical activity is based on the scientific exercise guidelines for adults with SCI [7], exercise guidelines of the American College of Sports Medicine [42] and the Dutch physical activity guidelines [43]. The information on healthy nutrition and energy expenditure is based on the guidelines and advices of the Dutch Nutrition Centre [44] and the brochure ‘Food, weight and health for people with SCI’ of the Swedish Spinalis Foundation [45], which was translated into Dutch in collaboration with the author (A-C Lagerström) and dieticians in the Dutch rehabilitation centers.

The WHEELS-app can be used stand-alone or guided. Individuals are in the position to plan existing exercise programs in their calendar, create own exercise programs and create their nutrition plans. Information and a format is shared with users on how to create an action plan. With guided use, the rehabilitation or lifestyle professional is assigned rights with which personal exercise programs and nutrition plans can be created and assigned to individuals by the rehabilitation or lifestyle professional to ensure correct choices of exercises and reasonable nutrition plans. The professional has access to the user profiles with progress tracking information and can involve individuals in lifestyle challenges for additional motivation. Communication with wheelchair users takes place via messages on their profile pages or e-mail. In addition, coaching can be done by phone.

After development, the individual parts of the app were pre-tested on ease of use and satisfaction in a user study among 24 wheelchair users and 5 rehabilitation professionals. Based on the results, minor adjustments were made with regard to the instruction and findability of the various parts of the app. Subsequently, the complete app was tested in a pilot study. The method and results of this pilot study are described and discussed later in this article.

### Step 5: program implementation plan

For the purpose of the pilot study, the WHEELS-app was implemented on a small scale via the two participating rehabilitation centers. Part of the implementation strategy was to involve wheelchair users and rehabilitation professionals in the development of the mobile lifestyle application. After processing the results of the pilot study, a strategy was developed for the national implementation of the WHEELS-app. Theory and empiric literature on the implementation of health promotion programs were consulted [32,46,47]. In addition, interviews were held with intermediate organizations with a close connection with wheelchair users and rehabilitation professionals to gain insight into the barriers and stimulating factors for successful adoption, implementation and maintenance of the WHEELS-app. The Measurement Instrument for Determinants of Innovations (MIDI) was used to prepare these interviews [46]. Based on the results, a plan was developed to ensure that the WHEELS-app would be adopted, implemented and maintained.

### Step 6: program evaluation plan

An evaluation plan was developed for the pilot study. This plan describes both the process and the effect evaluation. The process evaluation aimed to determine the usability and feasibility of the mobile lifestyle intervention. The effect evaluation aimed to assess whether the intervention has the desired effects on the lifestyle, health and quality of life of wheelchair users with SCI or LLA. After the intervention has been improved based on the results of the pilot study, a more extensive evaluation plan will be developed that can be used to evaluate the intervention after national implementation.

## Matrices of change objectives created in the WHEELS project to direct the development of a mobile lifestyle intervention for wheelchair users

| Matrix of change objectives (performance objectives linked to behavioral determinants) showing the steps that wheelchair users (WU) with a spinal cord injury or lower limb amputation should take to meet the physical activity and exercise guidelines. | | | | | |
| --- | --- | --- | --- | --- | --- |
| **Performance objectives** | **Determinants** | | | | |
|  | ***Attitude*** | ***Awareness*** | ***Self-efficacy and skills*** | ***Knowledge*** | ***Outcome expectations*** |
| **Precontemplation and contemplation stage** | | | | | |
| PO1. WU are acquainted with the applicable physical activity and exercise guidelines. | At1a. WU are positive about reading the applicable physical activity and exercise guidelines. | Aw1a. WU are aware of the existence of applicable physical activity and exercise guidelines. | SeS1a. WU are confident that they can find and understand the applicable physical activity and exercise guidelines.  SeS1b. WU show where they can find the applicable physical activity and exercise guidelines. | Kn1a. WU describe when they meet the applicable physical activity and exercise guidelines. | Oe1a. WU expect that knowledge of the applicable physical activity and exercise guidelines will help them to become physically active or start exercising. |
| **Preparation and action stage** | | | | | |
| PO2. WU give reasons for being physically active and exercise participation. |  | Aw2a. WU are aware of the importance of physical activity and exercise. |  | Kn2a. WU list the advantages of physical activity and exercise participation. | Oe2a. WU have realistic expectations regarding the benefits of physical activity and exercise participation. |
| PO3. WU decide to become (more) physically active and start exercising (more often). | At3a. WU have a positive attitude towards participation in physical activity and exercise activities. |  | SeS3a. WU have confidence that they will be able to be physically active and/or exercise (more often). |  | Oe3a. WU expect that participation in physical activity and exercise activities will have a positive effect on their health and well-being. |
| PO4. WU set goals and make their own physical activity and exercise plan. | At4a. WU have a positive attitude towards making an action plan. | Aw4a. WU are aware that setting goals and making an action plan helps them to become and stay physically active. | SeS4a. WU are confident that they can set realistic physical activity and exercise goals.  SeS4b. WU show their action plan. | Kn4a. WU name physical activity and exercise activities that match their goals and possibilities.  Kn4b. WU formulate SMART physical activity and exercise goals.  Kn4c. WU describe what an action plan must meet. | Oe4a. WU expect that setting goals and drawing up an action plan will help them to become and stay physically active and fit. |
| PO5. WU (who need it) ask help from their social environment to become and stay physically active. | At5a. WU have a positive attitude towards asking their social environment for help in becoming and staying physically active. | Aw5a. WU are aware of the will and possibilities of their social environment to help them become and stay physically active. | SeS5a. WU are confident that they can ask their social environment for help to become and stay physically active. |  | Oe5a. WU have realistic expectations of the help and support that their social environment can provide in becoming and staying physically active. |
| PO6. WU are acquainted with the physical activity and exercise possibilities in their living environment. | At6a. WU have a positive attitude towards exercise and physical activity close to home. | Aw6a. WU are aware that travel time and transport problems are barriers to becoming and staying physically active. | SeS6a. WU are confident that they can identify the exercise and physical activity options in their living environment.  SeS6b. WU show the sports and exercise options in their living environment. | Kn6a. WU show where they can find information about suitable exercise and physical activity options in their living environment.  Kn6b. WU name suitable sports activities that are offered in and around their place of residence. | Oe6a. WU expect that awareness of physical activity and exercise opportunities close to home will facilitate a physically active lifestyle. |
| PO7. WU are more physically active in everyday life. | At7a. WU have a positive attitude towards being more physically active in daily life. | Aw7a. WU are aware of their current level of physical activity.  Aw7b. WU are aware of moments in daily life when they can be more physically active. | SeS7a. WU are confident that they can overcome barriers and manage to be more physically active in everyday life. | Kn7a. WU name moments in everyday life when they can be more physically active.  Kn7b. WU describe how they can increase their level of physical activity in daily life. | Oe7a. WU expect that the incorporation of physical activity into daily life has a positive effect on their health and wellbeing. |
| PO8. WU train their cardiovascular fitness. | At8a. WU have a positive attitude towards improving their cardiovascular fitness. | Aw8a. WU are aware of the importance of good cardiovascular fitness. | SeS8a. WU are confident that they can train their cardiovascular fitness. | Kn8a. WU describe exercises and activities with which they can train their cardiovascular fitness. | Oe8a. WU expect that they can improve or maintain their cardiovascular fitness through exercise. |
| PO9. WU do muscle strengthening activities. | At9a. WU have a positive attitude towards muscle strengthening activities. | Aw9a. WU are aware of the importance of muscle strengthening activities. | SeS9a. WU are confident that they can train their muscle strength. | Kn9a. WU describe the ways in which they can train their muscle strength. | Oe9a. WU expect their muscle strength to improve or be maintained through exercise. |
| **Maintenance stage** | | | | | |
| PO10. WU have solutions for dealing with difficulties regarding exercise and physical activity. | At10a. WU have a positive attitude towards making a coping plan. | Aw10a. WU are aware of possible pitfalls in exercise and physical activity planning. | SeS10a. WU are confident that they will be able to keep exercising and stay physically active in difficult situations.  SeS10b. WU show their coping plan. | Kn10a. WU describe strategies to keep exercising regularly in difficult situations.  Kn10b. WU describe how to make a coping plan. | Oe10a. WU expect that making a coping plan will help them to become and stay physically active. |
| PO11. WU enjoy their exercise and physical activity routine. |  | Aw11a. WU are aware that fun helps to maintain an exercise and physical activity routine. | SeS11a. WU are confident that they can enjoy exercise and physical activity. | Kn11a. WU describe sports and exercise activities that they enjoy. | Oe11a. WU expect that they will better adhere to sports and exercise activities that they enjoy. |
| PO12. WU have made it a routine to be physically active in daily life. | At12a. WU have a positive attitude towards making an exercise routine. | Aw12a. WU are aware of the importance of regular physical activity and exercise. | SeS12a. WU are confident that they can make a routine of their exercise and physical activity behavior. |  | Oe12a. WU expect that regular exercise and physical activity will have a positive effect on their health. |

| Matrix of change objectives (performance objectives linked to behavioral determinants) showing the steps that wheelchair users (WU) with a spinal cord injury or lower limb amputation should take to obtain a healthy energy balance. | | | | | |
| --- | --- | --- | --- | --- | --- |
| **Performance objectives** | **Determinants** | | | | |
|  | ***Attitude*** | ***Awareness*** | ***Self-efficacy and skills*** | ***Knowledge*** | ***Outcome expectations*** |
| **Preparation and action stage** | | | | | |
| PO1.  WU decide to work on a healthier diet. | At1a. WU have a positive attitude towards a healthy diet. |  | EV1a. WU are confident that they can eat healthier. |  | Oe1a. WU expect that adopting a healthy diet will have a positive effect on their health and wellbeing. |
| PO2. WU choose healthy food products. | At2a. WU have a positive attitude towards healthy food products. |  | SeS2a. WU are confident that they can choose healthy food products in the store. | Kn2a. WU know what healthy food products are. | Oe2a. WU expect healthy food products to positively influence their health and wellbeing. |
| PO3. WU adjust their energy intake to their energy expenditure. | At3a. WU have a positive attitude towards monitoring their weight. | Aw3a. WU are aware of their changed energy expenditure as a result of their illness and wheelchair use. | SeS3a. WU are confident that they can find out their daily calorie needs.  SeS3b. WU are confident that they can adjust their energy intake to their energy expenditure.  SeS3c. WU show how they match their energy intake to their energy expenditure.  SeS3d. WU will consult a dietician if they need help adjusting their energy intake to their energy consumption. | Kn3a. WU describe how they can find out and calculate the energy value of foods.  Kn3b. WU describe how they match their energy intake to their energy consumption. | Oe3a. WU expect that matching their energy intake to their energy expenditure will have a positive effect on their health and wellbeing. |
| PO4. WU have a varied diet. | At4a. WU have a positive attitude towards a varied diet. | Aw4a. WU describe how varied their current diet is. | SeS4a. WU are confident that they can adopt a varied diet.  SeS4b. WU show that they can eat varied meals. | Kn4a. WU describe a healthy and varied diet. | Oe4a. WU expect a varied diet to positively affect their health and wellbeing. |
| PO5. WU have realistic expectations of the consequences of a healthier diet. |  | Aw5a. WU are aware that a healthier diet leads to weight loss or a healthier body composition only if their energy expenditure is higher than their energy intake. |  | Kn5a. WU describe the potential health consequences of a healthy and unhealthy diet.  Kn5b. WU list the conditions for healthy weight loss. |  |
| PO6. WU involve their social environment in adjusting their diet. |  | Aw6a. WU are aware of the influence of their social environment on their diet. | SeS6a. WU are confident that their social environment will support them in adjusting their diet. | Kn6a. WU describe how their social environment can support them in adjusting their diet. |  |
| **Maintenance stage** | | | | | |
| PO7. WU have made a habit of eating healthy. |  | Aw7a. WU are aware of situations in which it is difficult to continue eating healthy. | SeS7a. WU are confident that they can maintain a healthy diet.  SeS7b. WU show a coping plan. |  |  |

| Matrix of change objectives (performance objectives linked to behavioral determinants) showing the steps that wheelchair users (WU) with a spinal cord injury or lower limb amputation should take to obtain a healthy balance between exercise and relaxation | | | | | |
| --- | --- | --- | --- | --- | --- |
| **Performance objectives** | **Determinants** | | | | |
|  | ***Attitude*** | ***Awareness*** | ***Self-efficacy and skills*** | ***Knowledge*** | ***Outcome expectations*** |
| **Preparation and action stage** | | | | | |
| PO1. WU have good body awareness. | At1a. WU recognize the importance of body awareness.  At1b. WU are open to learning more about body awareness and coping with fatigue. | Aw1a. WU are aware of their body signals.  Aw1b. WU are aware of their body posture.  Aw1c. WU describe how they recognize fatigue. | SeS1a. WU are confident that they can develop good body awareness. | Kn1a. WU know what body awareness is and how they can develop it. | Oe1a. WU expect good body awareness to positively affect their health and wellbeing. |
| PO2. WU apply principles related to balancing physical demand/load and capacity. | At2a. WU have a positive attitude towards applying principles related to balancing physical demand/load and capacity. | Aw2a. WU are aware of what is demanded from their body and the capacity of their body. | SeS2a. WU are confident that they can apply principles related to balancing physical demand and capacity.  SeS2b. WU show that they can apply principles related to balancing physical demand and capacity.  SeS2c. WU show how they can determine their degree of fatigue. | Kn2a. WU name principles related to balancing physical load and capacity. | Oe2a. WU expect that applying principles related to balancing physical load and capacity can protect them from going beyond their physical limits. |
| PO3. WU have solutions for coping with stressful situations. | At3a. WU recognize that they experience (sometimes) stressful situations.  At3b. WU are willing to pay attention to coping with stressful situations. | Aw3a. WU are aware of situations that they experience as stressful. | SeS3a. WU are confident that they can influence stressful situations. | Kn3a. WU describe how they can make situations they experience as stressful less stressful. | Oe3a. WU expect that recognizing stressful situations and thinking about solutions will lead to less stress. |
| PO4. WU can relax. | At4a. WU are positive about relaxation exercises. | Aw4a. WU are aware of the amount of tension in their body.  Aw4b. WU are aware of the importance of rest and relaxation for the recovery of their body. | SeS4a. WU trust they can rest and relax.  SeS4b. WU show relaxation exercises. |  | Oe4a. WU expect that relaxation exercises have a positive effect on their health and wellbeing. |
| PO5. WU have healthy sleeping habits. | At5a. WU have a positive attitude towards developing and maintaining healthy sleeping habits. | Aw5a. WU are aware of the influence of sleep on exercise and dietary behavior. | SeS5a. WU are confident that they can develop healthy sleeping habits.  SeS5b. WU show that they can apply healthy sleeping habits. | Kn5a. WU describe habits that promote sleep. | Oe5a. WU expect healthy sleeping habits to positively influence their health and wellbeing. |

| Matrix of change objectives (performance objectives linked to behavioral determinants) showing the steps that rehabilitation or lifestyle professionals should take to continue providing lifestyle guidance to wheelchair users with spinal cord injury or lower limb amputation after the rehabilitation phase. | | | | | |
| --- | --- | --- | --- | --- | --- |
| **Performance objectives** | **Determinants** | | | | |
|  | ***Perceived barriers*** | ***Knowledge*** | ***Social support*** | ***Skills*** |  |
| PO1. Professionals provide lifestyle counselling aimed at promoting physical activity/exercise, healthy diet and rest/relaxation after the rehabilitation phase. | PB1a. Professionals anticipate barriers to the provision of lifestyle counselling to WU after the rehabilitation phase. |  | SS1a. Professionals motivate WU to be physically active, exercise, eat healthy and pay attention to rest/relaxation and healthy sleep habits. | S1a. Professionals show how they help WU to formulate and pursue personal lifestyle goals. |  |
| PO2. Professionals encourage WU to comply with the applicable physical activity and exercise guidelines. | PB2a. Professionals anticipate factors that prevent them from encouraging WU to comply with the applicable physical activity and exercise guidelines. | Kn2a. Professionals describe how WU can comply with the applicable physical activity and exercise guidelines. | SS2a. Professionals motivate WU to comply with the applicable physical activity and exercise guidelines. | S2a. Professionals show how they encourage WU to comply with the applicable physical activity and exercise guidelines. |  |
| PO3. Professionals encourage WU to develop and maintain a healthy energy balance. | PB3a. Professionals anticipate barriers that prevent them from encouraging WU to develop and maintain a healthy energy balance. | Kn3a. Professionals describe how WU can develop and maintain a healthy energy balance. | SS3a. Professionals motivate WU to develop and maintain a healthy energy balance. | S3a. Professionals show how they encourage WU to develop and maintain a healthy energy balance. |  |
| PO4. Professionals encourage WU to develop a healthy balance between exercise and rest/relaxation. | PB4a. Professionals anticipate barriers that prevent them from encouraging WU to develop a healthy balance between exercise and rest/relaxation. | Kn4a. Professionals describe how WU can develop a healthy balance between exercise and rest/relaxation. | SS4a. Professionals motivate WU to develop a healthy balance between exercise and rest/relaxation. | S4a. Professionals show how they stimulate WU to develop a healthy balance between exercise and rest/relaxation. |  |

## Behavior change methods and practical strategies used to achieve the intervention goals of the WHEELS project.

| **Table 1.** Behavior change methods and practical strategies that have been selected to change the **attitude** and **outcome expectations** of wheelchair users in order to achieve the change objectives, and with this the intervention goals. | | |
| --- | --- | --- |
| **Change objectives aimed at meeting the applicable physical activity and exercise guidelines** | **Behavior change method** | **Practical strategy** |
| At1a. WU are positive about reading the applicable physical activity and exercise guidelines.  Oe1a. WU expect that knowledge of the applicable physical activity and exercise guidelines will help them to become physically active or start exercising. | Persuasive communication (Elaboration Likelihood Model)^32,48^  Tailoring (Trans-Theoretical Model)^32,34^ | Written messages and an infographic providing information and arguments to become familiar with the physical activity and exercise guidelines for persons with a SCI or LLA. |
| Oe2a. WU have realistic expectations regarding the benefits of physical activity and exercise participation. | Self-monitoring and Feedback (Social Cognitive Theory) ^32,37^ | Monitoring and graphical presentation of progress on self-formulated physical activity and exercise goals. |
| At3a. WU have a positive attitude towards participation in physical activity and exercise activities.  Oe3a. WU expect that participation in physical activity and exercise activities will have a positive effect on their health and wellbeing. | Persuasive communication (Elaboration Likelihood Model) ^32,48^  Modeling (Social Cognitive Theory) ^32,37^ | Written messages providing information about the pros of a physically active lifestyle and exercise and cons of sedentary behavior.  Videos and/or quotes from role models demonstrating what physical activity and exercise have brought them.  Ability to exchange experiences about overcoming barriers and the benefits of physical activity and exercise in an online community. |
| At4a. WU have a positive attitude towards making an action plan.  Oe4a. WU expect that setting goals and drawing up an action plan will help them to become and stay physically active and fit. | Tailoring (Trans-Theoretical Model) ^32,37^  Facilitation (Social Cognitive Theory) ^32,37^ | Providing a tailored example of an action plan, and a format to facilitate creating your own action plan. |
| At5a. WU have a positive attitude towards asking their social environment for help in becoming and staying physically active.  Oe5a. WU have realistic expectations of the help and support that their social environment can provide in becoming and staying physically active. | Direct experience (Learning Theories)^32^  Modeling (Social Cognitive Theory)^32,37^ | Possibility to ask questions to fellow wheelchair users.  Ability to contact a lifestyle coach (when using the app blended).  Role models share how they got help and what it brought them. |
| At6a. WU have a positive attitude towards exercise and physical activity close to home.  Oe6a. WU expect that awareness of physical activity and exercise opportunities close to home will facilitate a physically active lifestyle. | Persuasive communication (Elaboration Likelihood Model)^32,48^  Facilitation (Social Cognitive Theory)^32,37^ | Written messages providing information and arguments to look for suitable exercise and physical activities in the residential environment.  Exercise database and tailored fitness work-outs that can be performed in the home environment.  Link to national disability sports finder website which enables searching for a sports club by area. |

| **Table 1.** Continued from previous page. | | |
| --- | --- | --- |
| **Change objectives aimed at meeting the applicable physical activity and exercise guidelines** | **Behavior change method** | **Practical strategy** |
| At7a. WU have a positive attitude towards being more physically active in daily life.  Oe7a. WU expect that the incorporation of physical activity into daily life has a positive effect on their health and wellbeing. | Modeling (Social Cognitive Theory)^32,37^  Persuasive communication (Elaboration Likelihood Model)^32,48^ | Videos and/or quotes from role models showing that being more physically active in daily life has brought them health benefits.  Written messages providing information and arguments to incorporate physical activity into daily life. |
| At8a. WU have a positive attitude towards improving their cardiovascular fitness.  Oe8a. WU expect that they can improve or maintain their cardiovascular fitness through exercise. | Persuasive communication (Elaboration Likelihood Model)^32,48^  Facilitation (Social Cognitive Theory)^32,37^  Tailoring (Trans-Theoretical Model)^32,34^ | Written messages providing information and arguments to train cardiovascular fitness.  Exercise database and tailored fitness work-outs including cardiovascular exercises for wheelchair users. |
| At9a. WU have a positive attitude towards muscle strengthening activities.  Oe9a. WU expect their muscle strength to improve or be maintained through exercise. | Persuasive communication (Elaboration Likelihood Model)^32,48^  Facilitation (Social Cognitive Theory)^32,37^  Tailoring (Trans-Theoretical Model)^32,34^ | Written messages providing information and arguments to train muscle strength.  Exercise database and tailored fitness work-outs including cardiovascular exercises for wheelchair users. |
| At10a. WU have a positive attitude towards making a coping plan.  Oe10a. WU expect that making a coping plan will help them to become and stay physically active. | Tailoring (Trans-Theoretical Model)^32,34^  Facilitation (Social Cognitive Theory)^32,37^ | Providing a tailored example of a coping plan, and a format to facilitate creating your own coping plan. |
| Oe11a. WU expect that they will better adhere to sports and exercise activities that they enjoy. | Modeling (Social Cognitive Theory)^32,37^  Persuasive communication (Elaboration Likelihood Model)^32,48^ | Videos and/or quotes from role models showing that it is easier to maintain a sports or exercise activity if you enjoy it.  Written messages providing arguments to be physically active in a way that suits you and that you enjoy. |
| At12a. WU have a positive attitude towards making an exercise routine.  Oe12a. WU expect that regular exercise and physical activity will have a positive effect on their health. | Modeling (Social Cognitive Theory)^32,37^  Persuasive communication (Elaboration Likelihood Model)^32,48^ | Videos and/or quotes from role models showing that regular physical activity has improved their fitness, health and wellbeing.  Providing information and arguments that support the importance of regular physical activity and exercise. |
| **Change objectives aimed at developing and maintaining a healthy energy balance** | **Behavior change method** | **Practical strategy** |
| At1a. WU have a positive attitude towards a healthy diet.  Oe1a. WU expect that adopting a healthy diet will have a positive effect on their health and wellbeing. | Persuasive communication (Elaboration Likelihood Model)^32,48^  Modeling (Social Cognitive Theory)^32,37^ | Written messages providing information and arguments for the importance of a healthy diet for wheelchair users.  Videos and/or quotes from role models who share positive experiences with adjusting their diet. |
| At2a. WU have a positive attitude towards healthy food products.  Oe2a. WU expect healthy food products to positively influence their health and wellbeing. | Direct experience (Learning Theories)^32^  Self-monitoring and Feedback (Social Cognitive Theory)^32,37^ | Providing personalized feedback on daily calorie and nutrient intake. |
| **Table 1.** Continued from previous page. | | |
| **Change objectives aimed at developing and maintaining a healthy energy balance** | **Behavior change method** | **Practical strategy** |
| At3a. WU have a positive attitude towards monitoring their weight.  Oe3a. WU expect that matching their energy intake to their energy expenditure will have a positive effect on their health and wellbeing. | Facilitation (Social Cognitive Theory)^32,37^  Persuasive communication (Elaboration Likelihood Model)^32,48^ | Monitoring and graphical presentation of the course of body weight.  Written messages providing information and arguments for pursuing a healthy body-mass index. |
| At4a. WU have a positive attitude towards a varied diet.  Oe4a. WU expect a varied diet to positively affect their health and wellbeing. | Persuasive communication (Elaboration Likelihood Model)^32,48^  Facilitation (Social Cognitive Theory)^32,37^ | Written messages providing information and arguments for adopting a varied diet.  Possibility to exchange healthy recipes. |
| **Change objectives aimed at developing and maintaining a healthy balance between exercise and rest/relaxation.** | **Behavior change method** | **Practical strategy** |
| At1a. WU recognize the importance of body awareness.  At1b. WU are open to learning more about body awareness and coping with fatigue.  Oe1a. WU expect good body awareness to positively affect their health and wellbeing. | Persuasive communication (Elaboration Likelihood Model)^32,48^  Facilitation (Social Cognitive Theory)^32,37^ | Written messages providing information and arguments for developing good body awareness.  Tips and tricks for developing body awareness and dealing with fatigue. |
| At2a. WU have a positive attitude towards applying principles related to balancing physical demand/load and capacity.  Oe2a. WU expect that applying principles related to balancing physical load and capacity can protect them from going beyond their physical limits. | Facilitation (Social Cognitive Theory)^32,37^  Modeling (Social Cognitive Theory)^32,37^ | Providing an explanation of the load and capacity model and practical tips to balance physical demand and capacity.  Videos and/or quotes from role models showing what balancing demand and capacity has brought them. |
| At3a. WU recognize that they experience (sometimes) stressful situations.  At3b. WU are willing to pay attention to coping with stressful situations.  Oe3a. WU expect that recognizing stressful situations and thinking about solutions will lead to less stress. | Active learning (Elaboration Likelihood Model)^32,37^  Persuasive communication (Elaboration Likelihood Model)^32,48^ | Exercise to identify factors that consume and provide physical and mental energy.  Written messages providing information and arguments for preventing stress to promote health. |
| At4a. WU are positive about relaxation exercises.  Oe4a. WU expect that relaxation exercises have a positive effect on their health and wellbeing. | Persuasive communication (Elaboration Likelihood Model)^32,48^  Facilitation (Social Cognitive Theory)^32,37^  Tailoring (Trans-Theoretical Model)^32,34^ | Written messages providing information and arguments to encourage doing relaxation exercises.  Providing relaxation exercises tailored for wheelchair users with SCI and LLA. |
| At5a. WU have a positive attitude towards developing and maintaining healthy sleeping habits.  Oe5a. WU expect healthy sleeping habits to positively influence their health and wellbeing. | Persuasive communication (Elaboration Likelihood Model)^32,48^ | Written messages and a brochure providing information, tips and arguments to adopt healthy sleeping habits. |

| **Table 2.** Behavior change methods and practical strategies that have been selected to change the **awareness** of wheelchair users in order to achieve the change objectives, and with this the intervention goals. | | |
| --- | --- | --- |
| **Change objectives aimed at meeting the applicable physical activity and exercise guidelines** | **Behavior change method** | **Practical strategy** |
| Aw1a. WU are aware of the existence of applicable physical activity and exercise guidelines. | Facilitation (Social Cognitive Theory)^32,37^ | Sharing a pdf and infographic of the physical activity and exercise guidelines for people with a SCI and people with a LLA. Additionally, the guidelines are summarized in short messages that are shared in an online community group around exercise and physical activity. |
| Aw2a. WU are aware of the importance of physical activity and exercise. | Personalize risk and benefits (Health Belief Model)^32,35^  Persuasive communication (Elaboration Likelihood Model)^32,48^ | Written messages providing information about personal costs or risks of sedentary behavior and arguments to exercise, become and stay physically active. |
| Aw4a. WU are aware that setting goals and making an action plan helps them to become and stay physically active. | Persuasive communication (Elaboration Likelihood Model)^32,48^ | Providing written information and arguments to set goals and create an action plan. |
| Aw5a. WU are aware of the will and possibilities of their social environment to help them become and stay physically active. | Consciousness raising (Trans-Theoretical Model)^32,34^  Persuasive communication (Elaboration Likelihood Model)^32,48^ | Written messages providing information about risks of not involving the social environment, and arguments to ensure social support in lifestyle change and the maintenance of healthy behavior.   - Short written messages in the online community. - In the face-to-face consultation at the start of the intervention attention is paid to the role that the social environment plays in behavioral change (only applies to blended use of the lifestyle app). |
| Aw6a. WU are aware that travel time and transport problems are barriers to becoming and staying physically active. | Consciousness raising (Trans-Theoretical Model)^32,34^ | Written messages containing information about the exercise barriers travel time and transport. Underlining the importance of looking for an exercise of physical activity in the residential area and the opportunity to exercise at home. |
| Aw7a. WU are aware of their current level of physical activity.  Aw7b. WU are aware of moments in daily life when they can be more physically active. | Self-monitoring and Feedback (Social Cognitive Theory)^32,37^  Consciousness raising (Trans-Theoretical Model)^32,34^ | Exercise and physical activity diary that provides insight into the daily exercise and physical activity pattern.  Possibility to link a wrist-worn activity monitor to the app.  Written messages providing information and examples of increasing physical activity during daily activities (tiny habits). |
| Aw8a. WU are aware of the importance of good cardiovascular fitness.  Aw9a. WU are aware of the importance of muscle strengthening activities. | Consciousness raising (Trans-Theoretical Model)^32,34^ | The exercise and physical activity guidelines for people with SCI and people with LLA are available through the lifestyle app. Additionally, the importance of cardiovascular fitness and muscle strengthening activities is explained in short written messages. |

| **Table 2.** Continued from previous page. | | |
| --- | --- | --- |
| **Change objectives aimed at meeting the applicable physical activity and exercise guidelines** | **Behavior change method** | **Practical strategy** |
| Aw10a. WU are aware of possible pitfalls in exercise and physical activity planning. | Consciousness raising (Trans-Theoretical Model)^32,34^  Persuasive communication (Elaboration Likelihood Model)^32,37^ | Written information and arguments to encourage identifying and anticipating factors that hinder physical activity and exercise. |
| Aw11a. WU are aware that fun helps to maintain an exercise and physical activity routine. | Consciousness raising (Trans-Theoretical Model)^32,34^ | Written messages providing information about the consequences of participating in unsuitable, unenjoyable exercise activities, and arguments to be physically active in a way that suits you and that you do enjoy. |
| Aw12a. WU are aware of the importance of regular physical activity and exercise. | Consciousness raising (Trans-Theoretical Model)^32,34^  Persuasive communication (Elaboration Likelihood Model)^32,48^ | Written messages providing information and arguments to exercise and to be physically active on a regular basis. Advise to plan activities using an action plan. |
| **Change objectives aimed at developing and maintaining a healthy energy balance** | **Behavior change method** | **Practical strategy** |
| Aw3a. WU are aware of their changed energy expenditure as a result of their illness and wheelchair use. | Consciousness raising (Trans-Theoretical Model)^32,34^  Personalize risk and benefits (Health Belief Model)^32,35^ | Written information about the consequences of SCI, LLA and a sedentary lifestyle on energy expenditure. |
| Aw4a. WU describe how varied their current diet is. | Consciousness raising (Trans-Theoretical Model)^32,34^  Personalize risk and benefits (Health Belief Model)^32,35^  Self-monitoring and Feedback (Social Cognitive Theory)^32,37^ | Written messages providing information about the importance of a varied diet and what a varied diet meets.  Possibility to keep a food diary. Based on this food diary, computer-tailored feedback is provided on the intake of carbohydrates, fats and proteins. |
| Aw5a. WU are aware that a healthier diet leads to weight loss or a healthier body composition only if their energy expenditure is higher than their energy intake. | Consciousness raising (Trans-Theoretical Model)^32,34^ | Written messages providing information about common causes of unsuccessful weight loss attempts, and conditions that must be met in order to lose weight/improve body composition successfully. |
| Aw6a. WU are aware of the influence of their social environment on their diet. | Consciousness raising (Trans-Theoretical Model)^32,34^  Persuasive communication (Elaboration Likelihood Model)^32,48^ | Written messages providing information about risks of not involving the social environment, and arguments to ensure social support in lifestyle change and the maintenance of healthy behavior.   - Short written messages in the online community. - In the face-to-face consultation at the start of the intervention attention is paid to the role that the social environment plays in behavioral change (only applies to blended use of the lifestyle app). |
| Aw7a. WU are aware of situations in which it is difficult to continue eating healthy. | Consciousness raising (Trans-Theoretical Model)^32,34^ | Written information and arguments to encourage identifying and anticipating factors that hinder healthy dietary behaviors. |
| **Table 2.** Continued from previous page. | | |
| **Change objectives aimed at developing and maintaining a healthy balance between exercise and rest/relaxation** | **Behavior change method** | **Practical strategy** |
| Aw1a. WU are aware of their body signals.  Aw1b. WU are aware of their body posture.  Aw1c. WU describe how they recognize fatigue. | Consciousness raising (Trans-Theoretical Model)^32,34^  Personalize risk and benefits (Health Belief Model)^32,35^ | Written information and a recorded presentation about body awareness, the causes and consequences of fatigue and recognizing physical and mental signs of fatigue.  Exercises to improve body awareness and body posture. |
| Aw2a. WU are aware of what is demanded from their body and the capacity of their body. | Consciousness raising (Trans-Theoretical Model)^32,34^  Personalize risk and benefits (Health Belief Model)^32,35^ | Written information and a recorded presentation about balancing body load/demand and capacity to reduce health problems and fatigue.  Exercise to gain insight into your own load-capacity balance. |
| Aw3a. WU are aware of situations that they experience as stressful. | Consciousness raising (Trans-Theoretical Model)^32,34^  Personalize risk and benefits (Health Belief Model)^32,35^ | Exercise to identify situations that consume and provide physical and mental energy. |
| Aw4a. WU are aware of the amount of tension in their body.  Aw4b. WU are aware of the importance of rest and relaxation for the recovery of their body. | Consciousness raising (Trans-Theoretical Model)^32,34^  Active learning (Elaboration Likelihood Model)^32,48^ | Written messages and a brochure providing information about the importance of adequate sleep and relaxation for recovery processes.  Exercises to improve body awareness. |
| Aw5a. WU are aware of the influence of sleep on exercise and dietary behavior. | Consciousness raising (Trans-Theoretical Model)^32,34^ | Written messages and brochure providing information about the causes and consequences of sleeping problems, and the importance of healthy sleeping habits. |

| **Table 3**. Behavior change methods and practical strategies that have been selected to change the **self-efficacy** and **skills** of wheelchair users in order to achieve the change objectives, and with this the intervention goals. | | | | |  |
| --- | --- | --- | --- | --- | --- |
| **Change objectives aimed at meeting the applicable physical activity and exercise guidelines** | **Behavior change method** | | **Practical strategy** | |  |
| SeS1a. WU are confident that they can find and understand the applicable physical activity and exercise guidelines.  SeS1b. WU show where they can find the applicable physical activity and exercise guidelines. | Facilitation (Social Cognitive Theory)^32,37^ | | Sharing a pdf and infographic of the physical activity and exercise guidelines for people with a SCI and people with a LLA. Additionally, the guidelines are summarized in short written messages that are shared in an online community group around exercise and physical activity. | |  |
| SeS3a. WU have confidence that they will be able to be physically active and/or exercise (more often). | Facilitation (Social Cognitive Theory)^32,37^  Modeling (Social Cognitive Theory)^32,37^ | | Exercise database and tailored fitness work-outs that are offered on three levels: beginner, intermediate and advanced.  Videos and/or quotes from role models showing that they had to overcome barriers to exercise or become (more) physically active, how they succeeded and how they manage to stay physically active. | |  |
| SeS4a. WU are confident that they can set realistic physical activity and exercise goals.  SeS4b. WU show their action plan. | Facilitation (Social Cognitive Theory)^32,37^  Goal setting (Goal-Setting Theory; Theories of Self-Regulation; Health Action Process Approach)^32,38,39^ | | Providing information and tailored examples of SMART goal setting.  Providing a tailored example of an action plan, and a format to facilitate creating your own action plan.  Presentation with voice recording explaining how to create an action plan. | |  |
| SeS5a. WU are confident that they can ask their social environment for help to become and stay physically active. | Planning coping responses (Theories of Self-Regulation; Health Action Process Approach)^32,39^ | | Exercise to list barriers to physical activity and exercise and to describe how the social environment can support in overcoming these barriers.  Online community group in which participants are challenged to share how their social environment supports them to participate in physical and exercise activities. | |  |
| SeS6a. WU are confident that they can identify the exercise and physical activity options in their living environment.  SeS6b. WU show the sports and exercise options in their living environment. | Facilitation (Social Cognitive Theory)^32,37^ | | Information about the role and accessibility of the neighborhood sports coach and sports consultant in adapted sports.  Link to national disability sports finder website which enables searching for a sports club by area, sports category, age category and type of impairment. | |  |
| SeS7a. WU are confident that they can overcome barriers and manage to be more physically active in everyday life. | Planning coping responses (Theories of Self-Regulation; Health Action Process Approach)^32,39^  Persuasive communication (Elaboration Likelihood Model)^32,48^ | | Exercise to list potential barriers to incorporating physical activities into daily life and ways to overcome these barriers.  Short written messages providing arguments to maintain a physically active lifestyle in the long term. | |  |
| SeS8a. WU are confident that they can train their cardiovascular fitness. | Set graded tasks (Social Cognitive Theory; Theories of Self-Regulation; Health Action Process Approach)^32,37,39^  Facilitation (Social Cognitive Theory)^32,37^  Tailoring (Trans-Theoretical Model)^32,34^ | | Exercise database and tailored fitness work-outs including cardiovascular exercises for wheelchair users. These exercises are offered on three levels: beginner, intermediate and advanced. A 3D-animated personal trainer shows the correct performance of each exercise. | |  |
| **Table 3.** Continued from previous page. | | | | |  |
| **Change objectives aimed at meeting the applicable physical activity and exercise guidelines** | **Behavior change method** | | **Practical strategy** | |  |
| SeS9a. WU are confident that they can train their muscle strength. | Set graded tasks (Social Cognitive Theory; Theories of Self-Regulation; Health Action Process Approach)^32,37,39^  Facilitation (Social Cognitive Theory)^32,37^  Tailoring (Trans-Theoretical Model)^32,34^ | | Exercise database and tailored fitness work-outs including muscle strengthening exercises for wheelchair users. These exercises are offered on three levels: beginner, intermediate and advanced and are suitable for people with more or less severely impaired arm and/or leg function. A 3D-animated personal trainer shows the correct performance of each exercise. | |  |
| SeS10a. WU are confident that they will be able to keep exercising and stay physically active in difficult situations.  SeS10b. WU show their coping plan. | Planning coping responses (Theories of Self-Regulation; Health Action Process Approach)^32,39^  Persuasive communication (Elaboration Likelihood Model)^32,48^  Facilitation (Social Cognitive Theory)^32,37^ | | Presentation with voice recording explaining how to overcome potential barriers by creating a coping plan.  Providing a tailored example of a coping plan, and a format to facilitate creating your own coping plan. | |  |
| SeS11a. WU are confident that they can enjoy exercise and physical activity. | Modeling (Social Cognitive Theory)^32,37^ | | Videos and/or quotes from role models showing that it is possible to find an enjoyable sports or exercise activity and what this has brought them. | |  |
| SeS12a. WU are confident that they can make a routine of their exercise and physical activity behavior. | Goal setting (Goal-Setting Theory; Theories of Self-Regulation; Health Action Process Approach)^32,38,39^  Self-monitoring and Feedback (Social Cognitive Theory)^32,37^ | | Calendar in which sports and exercise activities can be scheduled.  Exercise and physical activity diary that provides insight into the daily exercise and physical activity pattern.  Possibility to set exercise and physical activity goals and to keep track of the progress towards achieving these goals. | |  |
| **Change objectives aimed at developing and maintaining a healthy energy balance** | **Behavior change method** | | **Practical strategy** | |  |
| SeS1a. WU are confident that they can eat healthier. | Facilitation (Social Cognitive Theory)^32,37^  Modeling (Social Cognitive Theory)^32,37^  Self-monitoring and Feedback (Social Cognitive Theory)^32,37^ | | Providing a brochure and short written messages with information, tips and tricks regarding adopting and maintaining a healthy diet.  Possibility to keep a food diary. Based on this food diary, computer-tailored feedback is provided on the daily calorie intake and intake of carbohydrates, fats and proteins.  Providing computer-tailored meal plans based on personal goals.  Possibility to exchange healthy recipes.  Videos and/or quotes from role models showing that it is possible to adopt and maintain a healthy diet. They tell how they have overcome barriers and what it has brought them. | |  |
| SeS2a. WU are confident that they can choose healthy food products in the store. | Facilitation (Social Cognitive Theory)^32,37^ | | Barcode scanner and food product list providing insight into the calories and nutrients in food products. | |  |
| **Table 3.** Continued from previous page. | | | | | |
| **Change objectives aimed at developing and maintaining a healthy energy balance** | | **Behavior change method** | | **Practical strategy** | |
| SeS3a. WU are confident that they can find out their daily calorie needs.  SeS3b. WU are confident that they can adjust their energy intake to their energy expenditure.  SeS3c. WU show how they match their energy intake to their energy expenditure.  SeS3d. WU will consult a dietician if they need help adjusting their energy intake to their energy consumption. | | Facilitation (Social Cognitive Theory)^32,37^  Self-monitoring en Feedback (Social Cognitive Theory)^32,37^  Tailoring (Trans-Theoretical Model)^32,34^ | | Personalized advice regarding a healthy daily calorie intake.  Possibility to keep a food diary. Based on this food diary, computer-tailored feedback is provided on the daily calorie intake and intake of carbohydrates, fats and proteins.  Information about consulting and finding a dietician specializing in SCI or LLA. | |
| SeS4a. WU are confident that they can adopt a varied diet.  SeS4b. WU show that they can eat varied meals. | | Facilitation (Social Cognitive Theory)^32,37^  Self-monitoring en Feedback (Social Cognitive Theory)^32,37^  Persuasive communication (Elaboration Likelihood Model)^32,48^ | | Providing a brochure and short written messages with information, tips and tricks regarding adopting and maintaining a varied diet.  Barcode scanner and food product list providing insight into the nutrients in food products.  Possibility to exchange healthy recipes. | |
| SeS6a. WU are confident that their social environment will support them in adjusting their diet. | | Planning coping responses (Theories of Self-Regulation; Health Action Process Approach)^32,39^ | | Exercise to list barriers to adopting and a healthy diet and to describe how the social environment can support in overcoming these barriers.  Online community group in which participants are challenged to share how their social environment supports them to eat healthy. | |
| SeS7a. WU are confident that they can maintain a healthy diet.  SeS7b. WU show a coping plan. | | Planning coping responses (Theories of Self-Regulation; Health Action Process Approach)^32,39^  Persuasive communication (Elaboration Likelihood Model)^32,48^  Facilitation (Social Cognitive Theory)^32,37^ | | Presentation with voice recording explaining how to overcome potential barriers by creating a coping plan.  Providing a tailored example of a coping plan, and a format to facilitate creating your own coping plan.  Providing a brochure and short written messages with information, tips and tricks regarding adopting and maintaining a healthy diet. | |
| **Change objectives aimed at developing and maintaining a healthy balance between exercise and rest/relaxation** | | **Behavior change method** | | **Practical strategy** | |
| SeS1a. WU are confident that they can develop good body awareness. | | Facilitation (Social Cognitive Theory)^32,37^  Tailoring (Trans-Theoretical Model)^32,34^ | | Tailored exercises to improve body awareness and body posture. | |
| SeS2a. WU are confident that they can apply principles related to balancing physical demand and capacity.  SeS2b. WU show that they can apply principles related to balancing physical demand and capacity.  SeS2c. WU show how they can determine their degree of fatigue. | | Active learning (Elaboration Likelihood Model)^32,37^  Goal setting (Goal-Setting Theory; Theories of Self-Regulation; Health Action Process Approach)^32,38,39^ | | Information about applying the “ELCOSICO rules” (ELiminate, Change Order, Simplify, Combine) to save energy during activities and exercises to identify factors that consume and provide energy.  Possibility to plan daily activities and schedule time for rest and relaxation.  Information on how to use a numeric rating scale and BoWU scale to determine the degree of fatigue. | |
| **Table 3.** Continued from previous page. | | | | | |
| **Change objectives aimed at developing and maintaining a healthy balance between exercise and rest/relaxation** | | **Behavior change method** | | **Practical strategy** | |
| SeS3a. WU are confident that they can influence stressful situations. | | Persuasive communication (Elaboration Likelihood Model)^32,48^ | | Written messages providing information, tips and tricks on how to deal with stressful situations | |
| SeS4a. WU trust they can rest and relax.  SeS4b. WU show relaxation exercises. | | Facilitation (Social Cognitive Theory)^32,37^  Tailoring (Trans-Theoretical Model)^32,34^ | | Providing relaxation exercises tailored for wheelchair users with SCI and LLA. | |
| SeS5a. WU are confident that they can develop healthy sleeping habits.  SeS5b. WU show that they can apply healthy sleeping habits. | | Facilitation (Social Cognitive Theory)^32,37^  Tailoring (Trans-Theoretical Model)^32,34^ | | Written messages and a brochure tailored to wheelchair users with SCI or LLA providing information, tips and tricks on adopting healthy sleeping habits. | |

| **Table 4.** Behavior change methods and practical strategies that have been selected to change the **knowledge** of wheelchair users in order to achieve the change objectives, and with this the intervention goals. | | |
| --- | --- | --- |
| **Change objectives aimed at meeting the applicable physical activity and exercise guidelines** | **Behavior change method** | **Practical strategy** |
| Kn1a. WU describe when they meet the applicable physical activity and exercise guidelines. | Facilitation (Social Cognitive Theory)^32,37^  Persuasive communication (Elaboration Likelihood Model)^32,48^ | Link to PDF files of the scientific exercise guidelines for adults with spinal cord injury and Dutch physical activity guidelines.  Written messages and an infographic providing information and arguments to become familiar with the physical activity and exercise guidelines for persons with a SCI or LLA. |
| Kn2a. WU list the advantages of physical activity and exercise participation. | Persuasive communication (Elaboration Likelihood Model)^32,48^  Motivational Interviewing^32,40^ | Written messages providing information and arguments to exercise and be physically active.  When using the app blended, the lifestyle coach uses motivational interviewing to identify ambivalence to physical activity and exercise participation. |
| Kn4a. WU name physical activity and exercise activities that match their goals and possibilities.  Kn4b. WU formulate SMART physical activity and exercise goals.  Kn4c. WU describe what an action plan must meet. | Facilitation (Social Cognitive Theory)^32,37^  Persuasive communication (Elaboration Likelihood Model)^32,48^ | Providing information about sports and exercise activities for wheelchair users and the “Physical Activity Counseling Center” that can help to find a suitable sports and/or exercise activity.  Providing a tailored example of an action plan, and a format to facilitate creating your own action plan.  Presentation with voice recording explaining the importance of setting SMART goals, and how to create an action plan. |

| **Table 4.** Continued from previous page. | | |
| --- | --- | --- |
| **Change objectives aimed at meeting the applicable physical activity and exercise guidelines** | **Behavior change method** | **Practical strategy** |
| Kn6a. WU show where they can find information about suitable exercise and physical activity options in their living environment.  Kn6b. WU name suitable sports activities that are offered in and around their place of residence. | Facilitation (Social Cognitive Theory)^32,37^ | Information about the role and accessibility of the neighborhood sports coach and sports consultant in adapted sports.  Link to national disability sports finder website which enables searching for a sports club by area, sports category, age category and type of impairment. |
| Kn7a. WU name moments in everyday life when they can be more physically active.  Kn7b. WU describe how they can increase their level of physical activity in daily life. | Feedback (Social Cognitive Theory)^32,37^  Persuasive communication (Elaboration Likelihood Model)^32,48^ | Exercise and physical activity diary that provides insight into the daily exercise and physical activity pattern and moments when one could be more physically active.  Possibility to link a wrist-worn activity monitor to the app.  Written messages providing information and examples of increasing physical activity during daily activities (tiny habits). |
| Kn8a. WU describe exercises and activities with which they can train their cardiovascular fitness. | Facilitation (Social Cognitive Theory)^32,37^  Tailoring (Trans-Theoretical Model)^32,34^ | Written messages providing information and arguments to train cardiovascular fitness.  Exercise database and tailored fitness work-outs including cardiovascular exercises for wheelchair users. These exercises are offered on three levels: beginner, intermediate and advanced. A 3D-animated personal trainer shows the correct performance of each exercise. |
| Kn9a. WU describe the ways in which they can train their muscle strength. | Facilitation (Social Cognitive Theory)^32,37^  Tailoring (Trans-Theoretical Model)^32,34^ | Written messages providing information and arguments to train muscle strength.  Exercise database and tailored fitness work-outs including muscle strengthening exercises for wheelchair users. These exercises are offered on three levels: beginner, intermediate and advanced and are suitable for people with more or less severely impaired arm and/or leg function. A 3D-animated personal trainer shows the correct performance of each exercise. |
| Kn10a. WU describe strategies to keep exercising regularly in difficult situations.  Kn10b. WU describe how to make a coping plan. | Facilitation (Social Cognitive Theory)^32,37^  Motivational Interviewing^32,40^  Persuasive communication (Elaboration Likelihood Model)^32,48^ | Written information and arguments to encourage identifying and anticipating factors that hinder physical activity and exercise.  Videos and/or quotes from role models showing that they had to overcome barriers to exercise or become (more) physically active, how they succeeded and how they manage to stay physically active.  Presentation with voice recording explaining how to overcome potential barriers by creating a coping plan.  Providing a tailored example of a coping plan, and a format to facilitate creating your own coping plan. |

| **Table 4.** Continued from previous page. | | |
| --- | --- | --- |
| **Change objectives aimed at meeting the applicable physical activity and exercise guidelines** | **Behavior change method** | **Practical strategy** |
| Kn11a. WU describe sports and exercise activities that they enjoy. | Facilitation (Social Cognitive Theory)^32,37^ | Offering / getting acquainted with varied sports and exercise activities through an exercise database and referral to the Physical Activity Counseling Center. |
| **Change objectives aimed at developing and maintaining a healthy energy balance** | **Behavior change method** | **Practical strategy** |
| Kn2a. WU know what healthy food products are. | Facilitation (Social Cognitive Theory)^32,37^  Feedback (Social Cognitive Theory)^32,37^ | Providing a brochure and short written messages with information, tips and tricks regarding adopting and maintaining a healthy diet.  Providing personalized feedback on daily calorie and nutrient intake. |
| Kn3a. WU describe how they can find out and calculate the energy value of foods.  Kn3b. WU describe how they match their energy intake to their energy consumption. | Facilitation (Social Cognitive Theory)^32,37^  Feedback (Social Cognitive Theory)^32,37^ | Possibility to keep a food diary. Based on this food diary, computer-tailored feedback is provided on the daily calorie intake and intake of carbohydrates, fats and proteins.  Written information about the consequences of SCI, LLA and a sedentary lifestyle on energy expenditure.  Personalized advice regarding a healthy daily calorie intake. |
| Kn4a. WU describe a healthy and varied diet. | Facilitation (Social Cognitive Theory)^32,37^ | Providing a brochure and short written messages with information, tips and tricks regarding adopting and maintaining a healthy, varied diet. |
| Kn5a. WU describe the potential health consequences of a healthy and unhealthy diet.  Kn5b. WU list the conditions for healthy weight loss. | Facilitation (Social Cognitive Theory)^32,37^  Motivational Interviewing^32,40^ | Written messages providing:   - information and arguments for the importance of a healthy diet for wheelchair users. - information about common causes of unsuccessful weight loss attempts, and conditions that must be met in order to lose weight/improve body composition successfully.   When using the app blended, the lifestyle coach uses motivational interviewing to identify ambivalence to healthy food habits. |
| Kn6a. WU describe how their social environment can support them in adjusting their diet. | Modeling (Social Cognitive Theory)^32,37^ | Videos or written stories in which role models explain how their social environment has supported them in achieving a healthy diet. They tell how they got the support they need. |
| **Change objectives aimed at developing and maintaining a healthy balance between exercise and rest/relaxation** | **Behavior change method** | **Practical strategy** |
| Kn1a. WU know what body awareness is and how they can develop it. | Active learning (Elaboration Likelihood Model)^32,37^  Facilitation (Social Cognitive Theory)^32,37^ | Written messages providing information and tips for developing good body awareness.  Exercises to improve body awareness. |
| **Table 4.** Continued from previous page. | | |
| **Change objectives aimed at developing and maintaining a healthy balance between exercise and rest/relaxation** | **Behavior change method** | **Practical strategy** |
| Kn2a. WU name principles related to balancing physical load and capacity. | Active learning (Elaboration Likelihood Model)^32,37^  Facilitation (Social Cognitive Theory)^32,37^ | Written information and a recorded presentation about balancing body load/demand and capacity to reduce health problems and fatigue.  Exercise to gain insight into your own load-capacity balance. |
| Kn3a. WU describe how they can make situations they experience as stressful less stressful. | Facilitation (Social Cognitive Theory)^32,37^ | Written messages providing information, tips and tricks on how to deal with stressful situations |
| Kn5a. WU describe habits that promote sleep. | Facilitation (Social Cognitive Theory)^32,37^  Tailoring (Trans-Theoretical Model)^32,34^ | Written messages and a brochure tailored to wheelchair users with SCI or LLA providing information, tips and tricks on adopting healthy sleeping habits. |

| **Table 5.** Behavior change methods and practical strategies that have been selected to change **the perceived barriers** and **skills** of rehabilitation and lifestyle professionals in order to achieve the change objectives, and with this the intervention goals. | | |
| --- | --- | --- |
| **Change objective** | **Behavior change method** | **Practical strategy** |
| PB1a. Professionals anticipate barriers to the provision of lifestyle counselling to WU after the rehabilitation phase.  S1a. Professionals show how they help WU to formulate and pursue personal lifestyle goals. | Facilitation (Social Cognitive Theory)^32,37^  Guided practice (Social Cognitive Theory; Theories of Self-Regulation)^32,37^ | Lifestyle app with which rehabilitation and lifestyle professionals can support WU towards an active lifestyle.  Meeting in which rehabilitation and lifestyle professionals who use the app exchange experiences. |
| PB2a. Professionals anticipate factors that prevent them from encouraging WU to comply with the applicable physical activity and exercise guidelines.  S2a. Professionals show how they encourage WU to comply with the applicable physical activity and exercise guidelines. | Facilitation (Social Cognitive Theory)^32,37^  Guided practice (Social Cognitive Theory; Theories of Self-Regulation)^32,37^ | Exercise database and tailored fitness work-outs that can be used to improve the physical activity and fitness of wheelchair users.  Meeting in which rehabilitation and lifestyle professionals who use the app exchange experiences. |
| PB3a. Professionals anticipate barriers that prevent them from encouraging WU to develop and maintain a healthy energy balance.  S3a. Professionals show how they encourage WU to develop and maintain a healthy energy balance. | Facilitation (Social Cognitive Theory)^32,37^  Guided practice (Social Cognitive Theory; Theories of Self-Regulation)^32,37^ | Lifestyle app with which rehabilitation and lifestyle professionals can support WU towards a healthy energy balance.  Meeting in which rehabilitation and lifestyle professionals who use the app exchange experiences. |
| PB4a. Professionals anticipate barriers that prevent them from encouraging WU to develop a healthy balance between exercise and rest/relaxation.  S4a. Professionals show how they stimulate WU to develop a healthy balance between exercise and rest/relaxation. | Facilitation (Social Cognitive Theory)^32,37^  Guided practice (Social Cognitive Theory; Theories of Self-Regulation)^32,37^ | Lifestyle app with which rehabilitation and lifestyle professionals can support WU towards a healthy balance between exercise and rest/relaxation.  Meeting in which rehabilitation and lifestyle professionals who use the app exchange experiences. |

| **Table 6.** Behavior change methods and practical strategies that have been selected to change the **knowledge** of rehabilitation and lifestyle professionals in order to achieve the change objectives, and with this the intervention goals. | | |
| --- | --- | --- |
| **Change objective** | **Behavior change method** | **Practical strategy** |
| Kn2a. Professionals describe how WU can comply with the applicable physical activity and exercise guidelines. | Facilitation (Social Cognitive Theory)^32,37^ | Manual that describes how the app can be used to guide wheelchair users remotely to an active lifestyle.  Information on physical activity and exercise guidelines for wheelchair users.  Exercise programs that can be offered to wheelchair users.  Exercise database with which tailored exercise programs can be made.  Access to the exercise diary, exercise calendar and progress registration of WU. |
| Kn3a. Professionals describe how WU can develop and maintain a healthy energy balance. | Facilitation (Social Cognitive Theory)^32,37^ | Information about the energy expenditure (during rest and physical activities) of wheelchair users.  Manual that describes how the app can be used to guide wheelchair users remotely to a healthy energy balance.  Possibility to provide WU with nutritional advice via the app (advice contains information about calorie intake and distribution of nutrients) and to offer meal plans and recipes.  Possibility to provide WU with information about a healthy and varied diet through the app.  Digital food diary based on which feedback can be provided. |
| Kn4a. Professionals describe how WU can develop a healthy balance between exercise and rest/relaxation. | Facilitation (Social Cognitive Theory)^32,37^ | App that offers the possibility to provide WU with information about the development of a healthy balance between exercise and rest/relaxation (information about healthy sleeping habits and balancing load/demand and capacity).  Relaxation exercises that can be offered to WU through the app.  Possibility to gain insight into the exercise diary of WU so that insight can be obtained into moments when exercise and rest/relaxation are not in balance. |

| **Table 7.** Behavior change methods and practical strategies that have been selected to change **the social support** of rehabilitation and lifestyle professionals in order to achieve the change objectives, and with this the intervention goals. | | |
| --- | --- | --- |
| **Change objective** | **Behavior change method** | **Practical strategy** |
| SS1a. Professionals motivate WU to be physically active, exercise, eat healthy and pay attention to rest/relaxation and healthy sleep habits. | Participation (Organizational Development Theories)^32,49^  Facilitation (Social Cognitive Theory)^32,37^ | Rehabilitation professionals are included in the planning group that is developing a mobile lifestyle intervention for WU.  Rehabilitation and lifestyle professionals have an app at their disposal that they can use to remotely motivate wheelchair users to be physically active, exercise, eat healthy and pay attention to rest/relaxation and healthy sleep habits. |
| SS2a. Professionals motivate WU to comply with the applicable physical activity and exercise guidelines. | Participation (Organizational Development Theories)^32,49^  Facilitation (Social Cognitive Theory)^32,37^ | Rehabilitation professionals are included in the planning group that is developing the exercise module of a mobile lifestyle intervention for WU.  Rehabilitation and lifestyle professionals have an app at their disposal that they can use to remotely motivate wheelchair users to comply with the applicable physical activity and exercise guidelines. |
| SS3a. Professionals motivate WU to develop and maintain a healthy energy balance. | Participation (Organizational Development Theories)^32,49^  Facilitation (Social Cognitive Theory)^32,37^ | Rehabilitation professionals are included in the planning group that is developing the food module of a mobile lifestyle intervention for WU.  Dieticians working in the Dutch rehabilitation centers are involved in the development of the nutritional information materials.  Rehabilitation and lifestyle professionals have an app at their disposal that they can use to remotely motivate wheelchair users to develop and maintain a healthy energy balance. |
| SS4a. Professionals motivate WU to develop a healthy balance between exercise and rest/relaxation. | Participation (Organizational Development Theories)^32,49^  Facilitation (Social Cognitive Theory)^32,37^ | Rehabilitation professionals are included in the planning group that is developing the rest/relaxation module of a mobile lifestyle intervention for WU.  Rehabilitation and lifestyle professionals have an app at their disposal that they can use to remotely motivate wheelchair users to develop a healthy balance between exercise and rest/relaxation. |

## References

1. Green LW, Kreuter MW. Health Program Planning: An Educational and Ecological Approach. 4th ed. New York: McGraw-Hill Higher Education; 2005.

2. Van Den Berg-Emons RJ, Bussmann JB, Stam HJ. Accelerometry-based activity spectrum in persons with chronic physical conditions. Arch Phys Med Rehabil [Internet] Elsevier Inc.; 2010;91(12):1856–1861. PMID:21112426

3. Miller MJ, Jones J, Anderson CB, Christiansen CL. Factors influencing participation in physical activity after dysvascular amputation: a qualitative meta-synthesis. Disabil Rehabil [Internet] Taylor & Francis; 2019;41(26):3141–3150. PMID:30261758

4. RIVM. Beweegrichtlijnen [Internet]. 2019 [cited 2020 May 13]. Available from: https://www.sportenbewegenincijfers.nl/kernindicatoren/beweegrichtlijnen

5. RIVM. Sportdeelname wekelijks [Internet]. 2019 [cited 2020 May 13]. Available from: https://www.sportenbewegenincijfers.nl/kernindicatoren/sportdeelname-wekelijks%0D

6. Buman MP, Winkler EAH, Kurka JM, Hekler EB, Baldwin CM, Owen N, Ainsworth BE, Healy GN, Gardiner PA. Reallocating time to sleep, sedentary behaviors, or active behaviors: Associations with cardiovascular disease risk biomarkers, NHANES 2005-2006. Am J Epidemiol 2014;179(3):323–334. PMID:24318278

7. Martin Ginis KA, van der Scheer JW, Latimer-Cheung AE, Barrow A, Bourne C, Carruthers P, Bernardi M, Ditor DS, Gaudet S, de Groot S, Hayes KC, Hicks AL, Leicht CA, Lexell J, Macaluso S, Manns PJ, McBride CB, Noonan VK, Pomerleau P, Rimmer JH, Shaw RB, Smith B, Smith KM, Steeves JD, Tussler D, West CR, Wolfe DL, Goosey-Tolfrey VL. Evidence-based scientific exercise guidelines for adults with spinal cord injury: an update and a new guideline. Spinal Cord [Internet] Springer US; 2017;1–14. PMID:29070812

8. Beweegrichtlijn [Internet]. [cited 2020 Jun 26]. Available from: https://www.dwarslaesie.nl/dagelijks-leven/gezondheid/bewegen/

9. Lieberman J, Goff D, Hammond F, Schreiner P, Norton HJ, Dulin M, Zhou X, Steffen L. Dietary intake relative to cardiovascular disease risk factors in individuals with chronic spinal cord injury: A pilot study. Top Spinal Cord Inj Rehabil 2014;20(2):127–136. PMID:25477735

10. Tomey KM, Chen DM, Wang X, Braunschweig CL. Dietary intake and nutritional status of urban community-dwelling men with paraplegia. Arch Phys Med Rehabil 2005;86(4):664–671. [doi: 10.1016/j.apmr.2004.10.023] PMID:15827915

11. Westerkamp EA, Strike SC, Patterson M. Dietary intakes and prevalence of overweight/obesity in male non-dysvascular lower limb amputees. Prosthet Orthot Int 2019;43(3):284–292. [doi: 10.1177/0309364618823118] PMID:30663528

12. Giannoccaro MP, Moghadam KK, Pizza F, Boriani S, Maraldi NM, Avoni P, Morreale A, Liguori R, Plazzi G. Sleep disorders in patients with spinal cord injury. Sleep Med Rev [Internet] Elsevier Ltd; 2013;17(6):399–409. [doi: 10.1016/j.smrv.2012.12.005] PMID:23618534

13. Fogelberg DJ, Leland NE, Blanchard J, Rich TJ, Clark FA. Qualitative experience of sleep in individuals with spinal cord injury. OTJR Occup Particip Heal 2017;37(2):89–97. [doi: 10.1177/1539449217691978] PMID:28196449

14. Pell JP, Donnan PT, Fowkes FG, Ruckley C V. Quality of life following lower limb amputation for peripheral arterial disease. Eur J Vasc Surg 1993;7(4):448–451. PMID:8359304

15. Jensen MP, Truitt AR, Schomer KG, Yorkston KM, Baylor C, Molton IR. Frequency and age effects of secondary health conditions in individuals with spinal cord injury: A scoping review. Spinal Cord [Internet] Nature Publishing Group; 2013;51(12):882–892. [doi: 10.1038/sc.2013.112] PMID:24126851

16. Post MWM, Van Leeuwen CMC. Psychosocial issues in spinal cord injury: A review. Spinal Cord [Internet] Nature Publishing Group; 2012;50(5):382–389. [doi: 10.1038/sc.2011.182] PMID:22270190

17. Mckechnie PS, John A. Anxiety and depression following traumatic limb amputation: A systematic review. Injury [Internet] Elsevier Ltd; 2014;45(12):1859–1866. PMID:25294119

18. Nooijen CFJ, Vogels S, Bongers-Janssen HMH, Bergen MP, Stam HJ, Van Den Berg-Emons HJG. Fatigue in persons with subacute spinal cord injury who are dependent on a manual wheelchair. Spinal Cord [Internet] Nature Publishing Group; 2015;53(10):758–762. [doi: 10.1038/sc.2015.66] PMID:25896345

19. Wong S, Kenssous N, Hillier C, Pollmer S, Jackson P, Lewis S, Saif M. Detecting malnutrition risk and obesity after spinal cord injury: a quality improvement project and systematic review. Eur J Clin Nutr 2018;72(11):1555–1560. [doi: 10.1038/s41430-018-0194-y] PMID:29849183

20. Wen H, DeVivo MJ, Mehta T, Baidwan N, Chen Y. The impact of body mass index on one-year mortality after spinal cord injury. J Spinal Cord Med [Internet] Taylor & Francis; 2019; [doi: 10.1080/10790268.2019.1688021] PMID:31729925

21. Ravenek KE, Ravenek MJ, Hitzig SL, Wolfe DL. Assessing quality of life in relation to physical activity participation in persons with spinal cord injury: A systematic review. Disabil Health J [Internet] Elsevier Inc; 2012;5(4):213–223. [doi: 10.1016/j.dhjo.2012.05.005] PMID:23021731

22. Christensen J, Ipsen T, Doherty P, Langberg H. Physical and social factors determining quality of life for veterans with lower-limb amputation(s): a systematic review. Disabil Rehabil 2016;38(24):2345–2353. PMID:26985705

23. van den Akker LE, Holla JFM, Dadema T, Visser B, Valent LJ, de Groot S, Dallinga JM, Deutekom M. Determinants of physical activity in wheelchair users with spinal cord injury or lower limb amputation: perspectives of rehabilitation professionals and wheelchair users. Disabil Rehabil [Internet] Taylor & Francis; 2019;0(0):1–8. [doi: 10.1080/09638288.2019.1577503] PMID:30924706

24. Holla JFM, van den Akker LE, Dadema T, de Groot S, Tieland M, Weijs PJM, Deutekom M. Determinants of dietary behaviour in wheelchair users with spinal cord injury or lower limb amputation: Perspectives of rehabilitation professionals and wheelchair users. PLoS One 2020;15(1):1–19. [doi: 10.1371/journal.pone.0228465] PMID:32004359

25. Martin Ginis KA, Ma JK, Latimer-Cheung AE, Rimmer JH. A systematic review of review articles addressing factors related to physical activity participation among children and adults with physical disabilities. Health Psychol Rev 2016;10(4):478–494. [doi: 10.1080/17437199.2016.1198240] PMID:27265062

26. Kooijmans H, Post M, Motazedi E, Spijkerman D, Bongers-Janssen H, Stam H, Bussman H. Exercise self-efficacy is weakly related to engagement in physical activity in persons with long-standing spinal cord injury. Disabil Rehabil [Internet] Taylor & Francis; 2019;1–7. [doi: 10.1080/09638288.2019.1574914] PMID:30907149

27. Littman AJ, Boyko EJ, Thompson M Lou, Haselkorn JK, Sangeorzan BJ, Arterburn DE. Physical activity barriers and enablers in older Veterans with lower-limb amputation. 2014;51(6):895–906. [doi: 10.1682/JRRD.2013.06.0152] PMID:25356624

28. Martin Ginis KA, Latimer AE, Arbour-Nicitopoulos KP, Bassett RL, Wolfe DL, Hanna SE. Determinants of physical activity among people with spinal cord injury: A test of social cognitive theory. Ann Behav Med 2011;42(1):127–133. [doi: 10.1007/s12160-011-9278-9] PMID:21544701

29. Crawford DA, Hamilton TB, Dionne CP, Day JD. Barriers and facilitators to physical activity participation for men with transtibial osteomyoplastic amputation: A thematic analysis. J Prosthetics Orthot 2016;28(4):165–172. [doi: 10.1097/JPO.0000000000000109]

30. Phang SH, Martin Ginis KA, Routhier F, Lemay V. The role of self-efficacy in the wheelchair skills-physical activity relationship among manual wheelchair users with spinal cord injury. Disabil Rehabil 2012;34(8):625–632. [doi: 10.3109/09638288.2011.613516] PMID:21981243

31. Fernandez ME, ten Hoor GA, van Lieshout S, Rodriguez SA, Beidas RS, Parcel G, Ruiter RAC, Markham CM, Kok G. Implementation mapping: Using intervention mapping to develop implementation strategies. Front Public Heal 2019;7(JUN):1–15. [doi: 10.3389/fpubh.2019.00158] PMID:31275915

32. Bartholomew LK, Markham CM, Ruiter RAC, Fernandez ME, Kok G, Parcel GS. Planning health promotion programs: an intervention mapping approach. 4th ed. San Fransisco: Jossey-Bass; 2016.

33. Brug J, van Assema P, Lechner L. Gezondheidsvoorlichting en gedragsverandering: een planmatige aanpak. 9e ed. Assen: Koninklijke van Gorcum BV.; 2017.

34. Prochaska JO, Redding CA, Evers KE. The transtheoretical model and stages of change. In: Glanz K, Rimer BK, Viswanath K, editors. Heal Behav Heal Educ theory, Res Pract 4th ed San Fransisco, CA: Jossey-Bass; 2008. p. 98–120.

35. Champion VL, Skinner CS. The health belief model. In: Glanz K, Rimer BK, Viswanath K, editors. Heal Behav Heal Educ Theory, Res Pract 4th ed San Fransisco, CA: Jossey-Bass; 2008. p. 45–65.

36. Petty RE, Barden J, Wheeler SC. The elaboration likelihood model of persuasion: health promotions that yield sustained behavioral change. In: DiClemente RJ, Crosby RA, Kegler MC, editors. Emerg Theor Heal Promot Pract Res Strateg Improv public Heal 1st ed San Fransisco, CA: Jossey-Bass; 2002. p. 71–99.

37. McAlister AI, Perry CI, Parcel GS. How individuals, environments, and health behaviors interact: Social Cognitive Theory. In: Glanz K, Rimer BK, Viswanath K, editors. Heal Behav Heal Educ theory, Res Pract 4th ed San Fransisco, CA: Jossey-Bass; 2008. p. 169–187.

38. Latham GP, Locke EA. New developments in and directions for goal-setting research. Eur Psychol 2007;12(4):290–300.

39. Schwarzer R, Lippke S, Luszcynska A. Mechanisms of health behavior change in persons with chronic illness or disability: the Health Action Process Approach (HAPA). rehabilition Psychol 2011;56(3):161–170. PMID:21767036

40. Miller WR, Rollnick S. Motivational Interviewing: helping people change. 3rd ed. New York: The Guildford press; 2013.

41. Oinas-Kukkonen H, Harjumaa M. Persuasive systems design: Key issues, process model, and system features. Commun Assoc Inf Syst 2009;24(1):485–500. [doi: 10.17705/1cais.02428]

42. Riebe D, Ehman JK, Liguori G, Magal M, American College of Sports Medicine. ACSM’s guidelines for exercise testing and prescription. 10th ed. New York: Wolters Kluwer; 2018.

43. Health Council of the Netherlands. Dutch physical activity guidelines 2017. The Hague; 2017.

44. Voedingscentrum. Gezond eten [Internet]. [cited 2020 May 14]. Available from: https://www.voedingscentrum.nl/nl/gezond-eten-met-de-schijf-van-vijf.aspx

45. Lagerström A-C, Wahman K. The art of healthy living with physical impairments [Internet]. Available from: http://spinalis.se/wp-content/uploads/2015/05/The-art-of-healthy-living-with-physical-impairments.pdfISBN:9789186939588

46. Fleuren MAH, Paulussen TGWM, Dommelen P, Buuren S Van. Towards a measurement instrument for determinants of innovations. Int J Qual Heal Care 2014;26(5):501–510. [doi: 10.1093/intqhc/mzu060] PMID:24951511

47. Fernandez ME, Ruiter RAC, Markham CM, Kok G. Intervention mapping: Theory-and evidence-based health promotion program planning: Perspective and examples. Front Public Heal 2019;7(209). [doi: 10.3389/fpubh.2019.00209] PMID:31475126

48. Petty RE, Barden J, Wheeler SC. The elaboration likelihood model of persuasion: health promotions that yield sustained behavioral change. In: DiClemente RJ, Crosby RA, Kegler MC (Eds.), Emerging theories in health promotion practice and research: strategies for improving public health (p. 71-99). 1st ed. San Francisco, CA: Jossey-Bass. 2002.

49. Cummings TG, Worley CG. Organization Development & Change. 9th ed. Mason, OH: South-Western Cengage Learning. 2009.
