# Supplementary material for: Mobile App (WHEELS) to Promote a Healthy Lifestyle in Wheelchair Users With Spinal Cord Injury or Lower Limb Amputation: Usability and Feasibility Study
Source: JMIR Form Res. 2021 Aug 9;5(8):e24909. doi: 10.2196/24909 (PMC8386360; doi:10.2196/24909)
Supplement: Multimedia Appendix 2 [file formative_v5i8e24909_app2.docx]

## **Supplementary file 2.** Interview guide pilot study Wheelchair ExercisE and Lifestyle Study (WHEELS) project

| **Topics** | **Main questions** | **Possible follow-up questions** |
| --- | --- | --- |
| Opening | How experienced are you with using smartphone applications? | How long have you been using a smartphone?  Which lifestyle related apps do you already use?  For what reason do you use these apps? |
| Goals | What were your goals to work on using the WHEELS app? (e.g. exercising more often, eating healthier, more attention to relaxation) | What is the reason for working on these goals?  What was the reason for not working on goals in other lifestyle areas? |
| Adherence | To what extent have you used the WHEELS app for the entire 12 weeks? | How many times a week?  What did you use the WHEELS app for?  Which parts?  To what extent has the WHEELS app stimulated you to achieve your goals?  If not, when did you drop out?  What was the reason for that? |
| Motivation | What has (or has not) helped you to maintain your motivation to change your lifestyle? (personal factors and app related factors) | What went well in this?  What did you run into?  How did you manage to keep it up? |
| Ease of use | What came easy to you when it comes to using the app?  What was difficult for you when it comes to using the app? | Which aspects of the WHEELS app are unnecessary, could be improved, were unclear?  Which aspects of the WHEELS app are useful and clear?  Which parts did you expect in the app but were not included? |
| Satisfaction | What are you satisfied with regarding the app? | Can you give an example?  What did you run into that you were not satisfied with?  How did you solve that? |
| Usefulness | To what extent has the app influenced your exercise behavior and/or diet? | How has the app supported you in daily life in the past 12 weeks?  To what extent have you achieved your goals?  What do you think could be improved about the app? |
| Ending | Would you recommend the app to others? | Why?  Why not? |
