# Supplementary material for: Mobile App (WHEELS) to Promote a Healthy Lifestyle in Wheelchair Users With Spinal Cord Injury or Lower Limb Amputation: Usability and Feasibility Study
Source: JMIR Form Res. 2021 Aug 9;5(8):e24909. doi: 10.2196/24909 (PMC8386360; doi:10.2196/24909)
Supplement: Multimedia Appendix 3 [file formative_v5i8e24909_app3.docx]

| **Table S1.** Nutritional intake pre and post 12-week intervention based on diet records. | | | | | | | | | | |
| --- | --- | --- | --- | --- | --- | --- | --- | --- | --- | --- |
|  | Remote-guided (n=5) | |  | Stand-alone (n=5) | |  | All (n=10) | |  |  |
| Daily average consumed | Pre | Post | *P* value | Pre | Post | *P* value | Pre | Post | *P* value | Effect size |
| Kilocalories | 2085 ± 643 | 1478 ± 232 | .043^a^ | 1755 ± 392 | 1795 ± 452 | .69^a^ | 1920 ± 531 | 1637 ± 377 | .14^a^ | .46 |
| Protein (g) | 88.0 ± 16.0 | 70.2 ± 11.0 | .080^a^ | 71.6 ± 25.5 | 77.9 ± 15.8 | .23^a^ | 79.8 ± 21.8 | 74.1 ± 13.5 | .37 | .28 |
| Fat (g) | 86.0 ± 31.0 | 60.0 ± 17.1 | .043^a^ | 79.8 ± 35.1 | 79.1 ± 25.6 | .50^a^ | 82.9 ± 31.4 | 69.6 ± 22.9 | .074^a^ | .56 |
| Carbohydrates (g) | 198.6 ± 80.6 | 140.8 ± 44.6 | .043^a^ | 161.0 ± 32.6 | 168.5 ± 80.8 | .69^a^ | 179.8 ± 61.3 | 154.7 ± 63.3 | .24^a^ | .37 |
| Alcohol (g) | 17.5 ± 15.8 | 9.0 ± 12.4 | .14^a^ | 8.0 ± 6.8 | 6.8 ± 10.1 | .72^a^ | 12.7 ± 13.3 | 7.9 ± 10.7 | .16^a^ | .44 |
| Protein (%) | 17.5 ± 3.3 | 19.4 ± 4.5 | .23^a^ | 16.3 ± 4.4 | 17.7 ± 2.9 | .50^a^ | 16.9 ± 3.7 | 18.6 ± 3.6 | .14^a^ | .47 |
| Fat (%) | 36.7 ± 4.8 | 36.1 ± 6.7 | .69^a^ | 40.1 ± 11.5 | 40.2 ± 10.5 | .89^a^ | 38.4 ± 8.5 | 38.2 ± 8.6 | .88 | .05 |
| Carbohydrates (%) | 37.4 ± 5.8 | 37.7 ± 8.4 | .50^a^ | 37.9 ± 10.7 | 37.0 ± 12.9 | .89^a^ | 37.7 ± 8.1 | 37.3 ± 10.3 | .88^a^ | .05 |
| Alcohol (%) | 6.2 ± 5.7 | 4.7 ± 6.4 | .14^a^ | 2.7 ± 2.9 | 2.2 ± 3.2 | .47^a^ | 4.5 ± 4.7 | 3.4 ± 5.0 | .093^a^ | .53 |
| ^a^ Indicates non-parametric test used due to violation of normality with corresponding effect size. | | | | | | | | | | |

| **Table S2.** Body composition changes pre and post 12-week intervention. | | | | | | | | | | |
| --- | --- | --- | --- | --- | --- | --- | --- | --- | --- | --- |
|  | Remote-guided (n=6) | | | Stand-alone (n=7) | | | All (n=13) | |  |  |
| Body composition | Mean ± SD | | | Mean ± SD | | | Mean ± SD | |  |  |
|  | Pre | Post | *P* value | Pre | Post | *P* value | Pre | Post | *P* value | Effect size |
| Body mass (kg) | 89.2 ± 20.3 | 86.8 ± 22.9 | .12 | 77.7 ± 19.6 | 77.1 ± 18.9 | .24 | 83.0 ± 20.0 | 81.6 ± 20.6 | .091 | .49 |
| BMI (kg/m^2^) | 29.2 ± 5.7 | 28.3 ± 6.3 | .075 | 30.1 ± 9.7 | 29.9 ± 9.4 | .13 | 29.7 ± 7.8 | 29.1 ± 7.8 | .073 | .53 |
| WC (cm) | 106.1 ± 18.3 | 101.8 ± 19.5 | .028 | 102.1 ± 8.9 | 101.1 ± 8.1 | .24 | 103.9 ± 13.5 | 101.4 ± 13.8 | .015^b^ | .76 |
| FM (kg) | 35.4 ± 12.6 | 28.0 ± 12.7 | .028 | 29.4 ± 7.4 | 25.7 ± 5.8 | .13 | 32.1 ± 10.1 | 26.7 ± 9.2 | .004^b^ | .96 |
| FM (%) | 39.0 ± 7.2 | 31.2 ± 8.3 | .028 | 39.4 ± 11.7 | 35.4 ± 12.3 | .13 | 39.2 ± 9.5 | 33.4 ± 10.5 | .004^b^ | .97 |
| FFM (kg) | 53.9 ± 11.4 | 58.9 ± 13.1 | .046 | 48.3 ± 18.7 | 51.5 ± 21.4 | .18 | 50.9 ± 15.4 | 54.9 ± 17.7 | .023^b^ | .70 |
| FFM (%) | 61.0 ± 7.2 | 68.8 ± 8.3 | .028 | 60.6 ± 11.7 | 64.4 ± 12.3 | .13 | 60.8 ± 9.5 | 66.6 ± 10.5 | .004^b^ | .97 |
| ^b^ Indicates significant difference over time determined by a paired sample t-test (*P* < .05). Abbreviations: BMI = body mass index, WC = waist circumference, FM = fat mass, FFM = fat free mass. | | | | | | | | | | |

| **Table S3.** Results from questionnaires pre and post 12-week intervention. | | | | | | | | | | |  |
| --- | --- | --- | --- | --- | --- | --- | --- | --- | --- | --- | --- |
|  | | Remote-guided (n=4) | |  | Stand-alone (n=8) | |  | All (n=12) | |  |  |
|  | | Mean ± SD | |  | Mean ± SD | |  | Mean ± SD | |  |  |
| Questionnaire | | Pre | Post | *P* value | Pre | Post | *P* value | Pre | Post | *P* value | Effect size |
| PASIPD (MET h/day) | | 28.5 ± 18.4 | 26.58 ± 13.4 | 1.00 | 21.5 ± 14.0 | 20.2 ± 11.9 | .78 | 23.8 ± 15.1 | 22.4 ± 12.2 | .64 | .14 |
| GSES | | 31.8 ± 5.6 | 32.5 ± 4.8 | .28 | 35.3 ± 3.4 | 34.1 ± 4.1 | .53 | 34.1 ± 4.3 | 33.6 ± 4.2 | .69 | .11 |
| ESES | | 31.5 ± 4.3 | 29.8 ± 5.9 | .36 | 33.9 ± 3.6 | 33.8 ± 3.2 | .72 | 33.0 ± 3.8 | 32.4 ± 4.4 | .50^a^ | .07 |
| CIS20R | | 77.0 ± 15.3 | 72.0 ± 12.8 | .11 | 70.0 ± 9.6 | 68.4 ± 4.2 | .58 | 72.3 ± 11.6 | 69.6 ± 7.7 | .18^a^ | .39 |
| PSQI | | 7.8 ± 3.2 | 6.5 ± 2.6 | .10 | 8.1 ± 2.7 | 6.8 ± 2.2 | .18 | 8.0 ± 2.7 | 6.7 ± 2.2 | .063 | .57 |
| **SF-36E** | |  |  |  |  |  |  |  |  |  |  |
|  | Physical functioning | 43.8 ± 16.5 | 47.5 ± 8.7 | .46 | 56.3 ± 19.2 | 50.0 ± 22.0 | .35 | 52.1 ± 18.6 | 49.2 ± 18.2 | .58 | .16 |
|  | Social functioning | 68.8 ± 7.2 | 78.1 ± 15.7 | .18 | 75.0 ± 21.1 | 79.7 ± 24.0 | .60 | 72.9 ± 17.5 | 79.2 ± 20.9 | .36^a^ | .27 |
|  | Role limitation physical | 53.1 ± 19.4 | 48.4 ± 14.8 | .71 | 60.2 ± 29.9 | 64.8 ± 17.7 | .80 | 57.8 ± 26.1 | 59.4 ± 18.0 | .84 | .06 |
|  | Role limitation emotional | 66.7 ± 26.4 | 70.8 ± 19.8 | .85 | 85.4 ± 21.2 | 76.0 ± 28.0 | .18 | 79.2 ± 23.7 | 74.3 ± 24.7 | .53^a^ | .18 |
|  | Mental health | 82.5 ± 15.0 | 73.8 ± 13.8 | .10 | 81.3 ± 12.5 | 75.0 ± 16.7 | .55 | 81.7 ± 12.6 | 74.6 ± 15.1 | .13 | .46 |
|  | Energy/vitality | 59.4 ± 13.0 | 56.3 ± 8.8 | .41 | 64.1 ± 15.9 | 65.6 ± 12.9 | .94 | 62.5 ± 14.6 | 62.5 ± 12.2 | 1.00 | .00 |
|  | Pain | 59.2 ± 19.9 | 56.6 ± 23.9 | 1.00 | 58.9 ± 17.8 | 45.4 ± 20.8 | .046 | 59.0 ± 17.6 | 49.1 ± 21.5 | .11 | .48 |
|  | General health perceptions | 55.0 ± 18.3 | 50.0 ± 16.8 | .46 | 68.1 ± 17.5 | 65.0 ± 22.5 | .55 | 63.8 ± 18.1 | 60.0 ± 21.3 | .37^a^ | .26 |
| ^a^ Indicates non-parametrical test used due to violation of normality.  Abbreviations: PASIPD = Physical Activity Scale for individuals with Physical Disability, GSES = General Self-Efficacy Scale, ESES = Exercise Self-Efficacy Score, CIS20R = Checklist Individual Strength, PSQI = Pittsburgh Sleep Quality Index, SF36E = Short Form Health Survey 36. | | | | | | | | | | | |
